# Aerodynamic performance enhancement of centrifugal compressor using numerical techniques

**DOI:** 10.12688/f1000research.145060.1

**Published:** 2024-05-17

**Authors:** Shivani S, Amar Murthy A, Srinivas G

**Affiliations:** 1 Department of Mechanical & Industrial Engineering, Manipal Institute of Technology, Manipal Academy of Higher Education, Manipal, Karnataka, 576104, India; 2 Department of Aeronautical & Automobile Engineering, Manipal Institute of Technology, Manipal Academy of Higher Education, Manipal, Karnataka, 576104, India

**Keywords:** Centrifugal Compressor, ANSYS CFX, CFD, Turbulence, Optimization

## Abstract

**Background:**

Centrifugal compressors are dynamic machines utilizing a rotating impeller, efficiently accelerate incoming gases, transforming kinetic energy into pressure energy for compression. They serve a wide range of industries, including air conditioning, refrigeration, gas turbines, industrial processes, and applications such as air compression, gas transportation, and petrochemicals, demonstrating their versatility. Designing a centrifugal compressor poses challenges related to achieving high aerodynamic efficiency, surge and choke control, material selection, rotor dynamics, cavitation, erosion, and addressing environmental considerations while balancing costs. Optimizing maintenance, reliability, and energy efficiency are essential aspects of the design process.

**Methods:**

The primary objective of this research is to comprehensively investigate and improve the aerodynamic performance of centrifugal compressors. To accomplish this, a comprehensive investigation of variables such as blade number and hub diameter, along with various turbulence models will be conducted. This approach will leverage numerical techniques to fill the significant gaps in the current literature regarding centrifugal compressor design and optimization. The study encompasses the evaluation of two turbulence models, namely Shear Stress Transport and K-epsilon. Furthermore, it delves into the fine-tuning of blade geometry, including variations in blade number and hub diameter, aiming to refine the design for optimal performance. Extensive analyses using Ansys CFX encompass key variables such as Pressure, Mach Number, Density, Velocity, Turbulence Kinetic Energy, and Temperature.

**Results:**

Notably, the optimized pressure profile yielded remarkable results, achieving a substantial 36% improvement, demonstrating the tangible benefits of these design enhancements.

**Conclusion:**

The outcomes of this research hold significant utility for engineers, manufacturers, and regulatory bodies, offering invaluable insights and guidance to enhance compressor performance and efficiency.

## Introduction

The centrifugal compressor is a dynamic compressor with a radial design. It is used to compress gases by converting kinetic energy into pressure energy by using rotating parts to impart energy to the gas as it continuously flows. The main components of a centrifugal compressor are the casing, impeller, diffuser, bearings, seals, and the drive system. The impeller is the rotary part, and it has three main parts: vanes, hub, and shaft. During the working of the compressor, the gas is drawn into the centre of the impeller. As the impeller rotates rapidly, it induces the gas to spiral outward, acquiring kinetic energy in the process. The conversion of kinetic energy to pressure energy occurs when the gas flows through the diffuser, i.e., the stationary component of the compressor. The casing encloses the impeller and the diffuser of the compressor while directing the flow of gas. The centrifugal compressor is comparatively more efficient than other types of compressors as it requires less energy to operate. It can handle a high flow rate of gas, thus making it ideal for applications such as air conditioning and refrigeration. Since centrifugal force is not highly effective at high pressure, the centrifugal compressor is not suitable for high-pressure applications.

Centrifugal compressors have undergone many recent innovations aimed at enhancing their effectiveness and performance such as the use of advanced materials like composites and ceramics, digitalization for more precise monitoring, and implementation of adaptive control mechanisms. Improving the aerodynamic design of the compressor leads to better efficiency by reducing turbulence and friction in the flow of gas. The use of Computational Fluid Dynamics (CFD) has been widely used recently to simulate the flow and identify areas of improvement. The future scope of centrifugal compressors will most certainly be shaped by technological breakthroughs, changes in industrial requirements, and a growing emphasis on sustainability and efficiency. Considering the growing demand for portable devices, electronics cooling, and medical applications, the development of smaller and more compact centrifugal compressors could become a major topic of research. Additive manufacturing (3D printing) advancements may enable the fabrication of complicated geometries and customized components, resulting in enhanced compressor efficiency and performance.

## Literature review and objectives

### Literature on numerical methods

The advancement of technology made the use of numerical methods much easier than it was two decades back. The recent shift to simulations for understanding the areas of improvement led to more use of CFD for centrifugal compressors. In the past decade, many researchers have worked on understanding what is the variation from the experimental results to the numerical results; for this, many have made use of ANSYS CFX, FLUENT, MATLAB, OPEN FOAM, and open-source libraries. The findings in the research revealed discrepancies between the CFD conclusions and the experimental data. The differences vary depending on the operating conditions, such as at higher rotational speeds, turbulence models, mesh parameters, etc. ^1^^–^^3^ The authors speculated that the disparities could be attributed to the absence of key components in the CFD model. Further, understanding surge and preventing it by utilizing CFD is widely implemented. ^4^^–^^7^

The emergence of additive manufacturing and nanotechnology has reshaped the landscape, ushering in a new frontier in the development of centrifugal compressors—namely, the creation of microturbines optimized for enhanced portability. The pre-manufacturing design and testing of these micro models assumes paramount importance. In an influential work, Aghaei (2007) engaged in a multifaceted approach involving a 1D design using Centrifugal Compressor Design (CCD) code and subsequent CFD analysis for a 3D model featuring a centrifugal impeller with an approximate pressure ratio of 4:1. ^8^ The CFD analysis for the compressor is not restricted to the impeller but can also be used for analysis of inlet guide vanes, diffusers, and other parts of the compressor. ^9^^,^^10^ An equally consequential development pertains to the optimization of design, which has been substantially expedited due to the reduced resources required for generating new Computer-Aided Design (CAD) models in contrast to fully realized experimental prototypes. The optimization can be done in two ways: by manual method, and by parameterized design changes. Most of the optimizations were carried out by changing design parameters, such as trailing edge angles, polar angles, impeller shape factor, radius, blade height, and other design parameters. ^11^^–^^14^

### Literature on theoretical methods

The theoretical modeling of centrifugal compressors involves the creation of mathematical frameworks and computational simulations to elucidate their performance characteristics and behaviour. These models integrate fundamental principles of fluid dynamics, thermodynamics, and mechanical engineering to predict variables such as pressure ratios, flow rates, efficiency, and temperature distributions. Theoretical analyses encompass a range of factors, including impeller geometry, inlet conditions, and rotational speeds, allowing engineers to explore the compressor’s response under different operating scenarios. When compared to experimental methods, numerical simulation methods can give a more cost-effective and reliable alternative to researching the Tip Leakage Vortex (TLV). Different loss models are employed to forecast and assess the centrifugal compressor’s performance under varying circumstances and at various speeds. The study of various compressor losses, including leakage loss and recirculation loss, led to the development of models to calculate these losses. ^15^^–^^17^ Moreover, a novel model for calculating the volute loss accounts for the radial velocity loss, circumferential velocity loss, and wall friction loss. Compared to conventional semiempirical models, the model seeks to increase the accuracy of volute loss prediction. Total pressure losses, which are mostly produced in the inlet duct and inlet section, have an impact on the flow in a volute. The volute’s friction also contributes to overall pressure losses. ^16^^,^^18^^,^^19^

Reynolds-Averaged Navier-Stokes (RANS) stands out as a widely embraced CFD approach used to forecast fluid flow behaviour. It achieves this by solving the Navier-Stokes equations, which encompass viscosity and turbulence effects. These equations describe the motion of fluid particles and their interactions in a flow field. While being a well-known theoretical framework for the design and performance study of centrifugal compressors, the Aungier model was created by Dr. Ronald H. Aungier. This model serves as a useful tool for preliminary design and performance estimate since it offers a streamlined one-dimensional method for predicting the behaviour of centrifugal compressors. There are some variations between the steady-state RANS model and the 1D Aungier model, but both offer reasonable estimates of centrifugal compressor performance. ^20^ A theoretical framework used to explain the surge phenomenon in centrifugal and axial flow compressors is known as the Greitzer model, after Dr. Emory Leon Greitzer. The Greitzer approach is founded on the idea of feedback between the compressor’s flow rate and pressure. When adopting a relaxation time shorter than the one suggested for axial compressors, the model predictions and the experimental data are in a reasonable amount of agreement. ^21^

### Literature on experimental methods

Experimental analysis of centrifugal compressors involves conducting physical tests on prototypes, measuring their performance, and understanding their behaviour. This process often entails constructing and assembling actual compressor components and gathering data on parameters such as pressure ratios, flow rates, efficiency, temperature distributions, and surge behaviour. These experiments provide insights into how the compressor operates and validate theoretical models. By comparing results with numerical predictions, researchers can refine their understanding of factors contributing to discrepancies. Conducting both experimental and theoretical studies is essential for comprehending the impact of external factors on centrifugal compressors. An elevation in humidity, for instance, correlates with a reduction in the total pressure ratio. Introducing wet compression, however, enhances the total pressure ratio and isothermal efficiency, concurrently lowering the outlet total temperature and specific compression work. Encouragingly, the alignment between experimental and theoretical analyses concerning the influence of wet compression and humidity on centrifugal compressors underscores the validity of these findings. ^22^^,^^23^

Surge is a serious issue that continues to be the subject of a lot of research since it can harm the compressor and the system to which it is linked. When the flow rate through the compressor falls below the level at which the impellers can no longer sustain the necessary pressure rise, a surge occurs. As a result, the flow may change direction and the compressor may begin to vibrate erratically. The characteristics and behaviours of instability phenomena such as Rotating Instability (RI), impeller stall, and diffuser stall are closely associated with surge. ^24^ Casing treatments are found to reduce surge and reduce the noise of the compressor operating near surge. ^25^^,^^26^ Furthermore, on an unsteady flow field of a centrifugal compressor under pulsating backpressure conditions highly unsteady conditions can trigger the dynamic stall of the compressor, shifting the surge line to smaller mass flow rates ^27^^,^^28^

In addition, experiments were conducted to understand the influence of Adjustable Inlet Guide Vanes (AIGVs) and Adjustable Vaned Diffusers (AVDs) on the compressors’ pressure ratio, efficiency, and overall performance. It is found that the use of AIGVs and AVDs can significantly improve the compressors’ overall performance over the entire operating range. ^29^^,^^30^ Joo (2022) conducted an experimental study on the performance of supercritical CO _2_ centrifugal compressors with reference to the earlier available numerical simulations of the same. For the experimental study, a compressor performance test was conducted by controlling the rotational speeds of the impeller and the Calorific Value (CV) and further studied the effect of the change in the opening rate of the CV as well as the effect on the change in rotational speed of the impeller to draw the proper conclusion. ^31^

### Research gap

The literature review revealed a significant gap in research regarding the comprehension and enhancement of centrifugal compressors by investigating the influence of blade number and hub diameter variations on parameters such as pressure profile, Mach number, and eddy viscosity. Also, it was found that fewer studies have been conducted on various turbulence models which are available.

### Objective of research

To improve the aerodynamic performance enhancement of centrifugal compressor using numerical techniques.

## Methods

**Figure 1.**
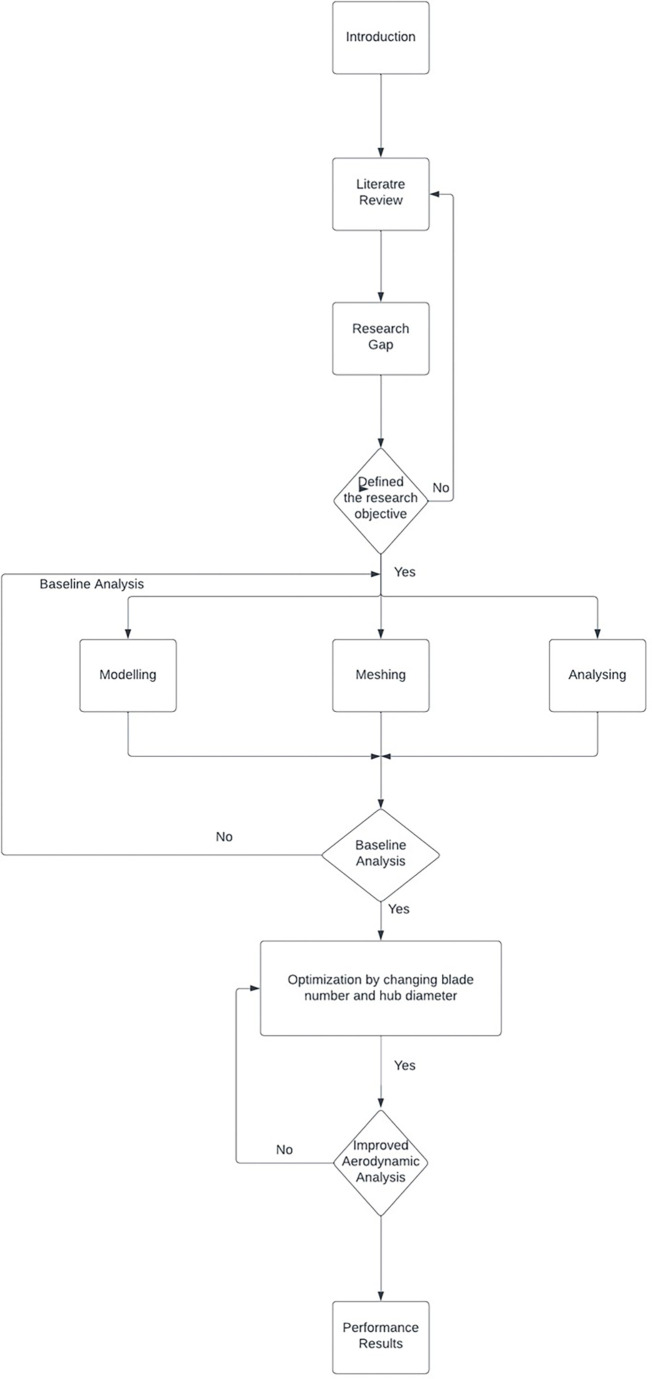
Methodology flow chart.

The research process for a paper on aerodynamic analysis commences with an introductory phase followed by an extensive literature review to discern prevailing knowledge and pinpoint research gaps. Subsequently, the research objectives are formulated, leading to the pivotal phase of conducting a baseline analysis, which establishes a fundamental benchmark, either through pre-existing data utilization or by performing rudimentary analyses. The subsequent stages encompass modeling and meshing, entailing the construction of a computational model and its division into discrete elements (mesh). Following this, an in-depth analysis of the model is conducted, employing computational simulations to elucidate airflow patterns around the subject of study. The outcomes of this analysis facilitate the identification of areas ripe for enhancement in aerodynamic performance. Researchers may then proceed to compile a comprehensive report and submit their findings for publication. Nevertheless, an iterative feedback loop underscores the process, allowing for refinement of both the model and mesh should the analysis yield unsatisfactory results. In essence, this comprehensive research process chart shown in Figure 1, rooted in the realm of aerodynamic analysis, encapsulates a methodical and iterative approach to scientific inquiry.

## Modeling of centrifugal compressor

**Figure 2.**
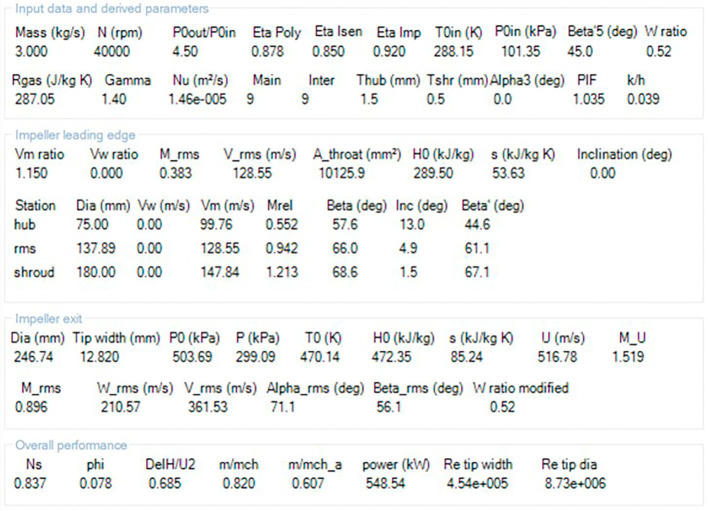
Results of the blade profile generated in Vista CCD.

**Figure 3.**
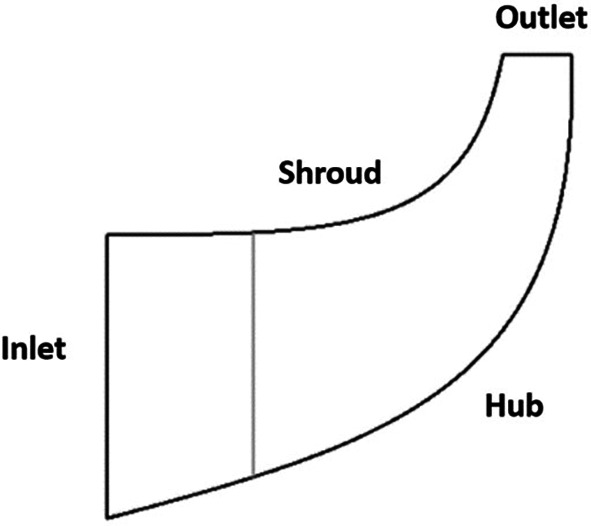
Impeller sketch.

**Figure 4.**
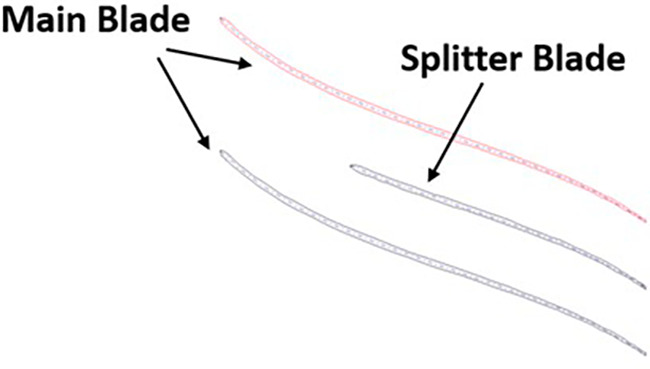
Blade-to-blade view.

**Figure 5.**
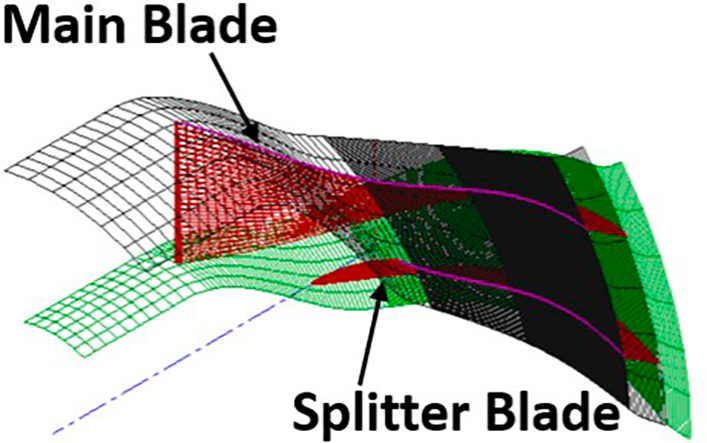
3D view of the blade.

**Figure 6.**
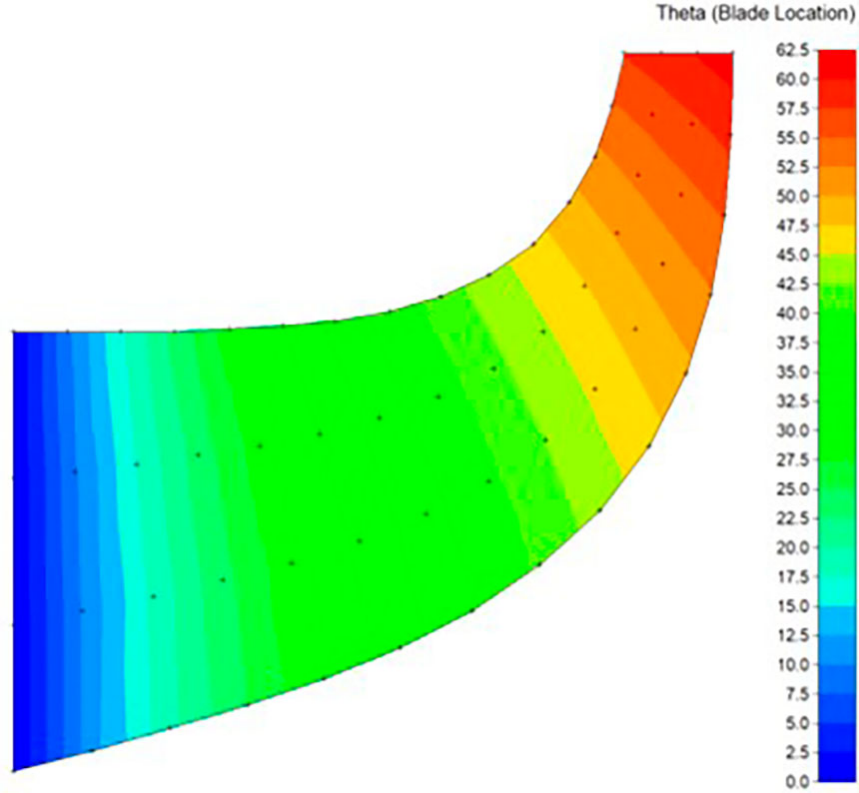
Meridional contour view.

**Figure 7.**
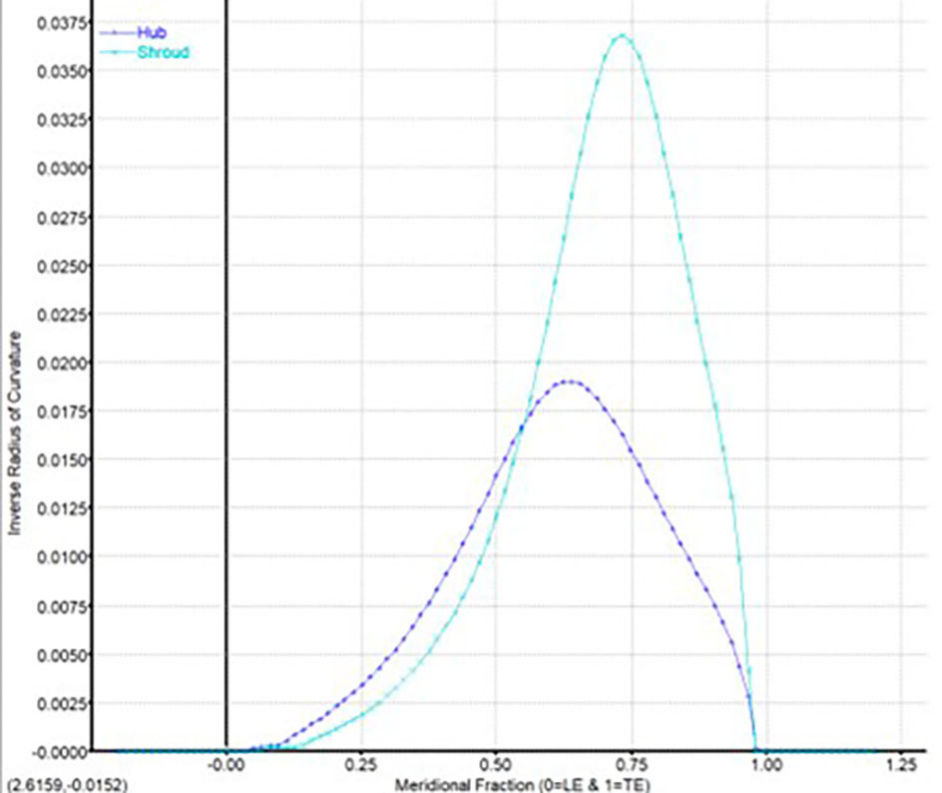
Meridional curvature graph.

**Figure 8.**
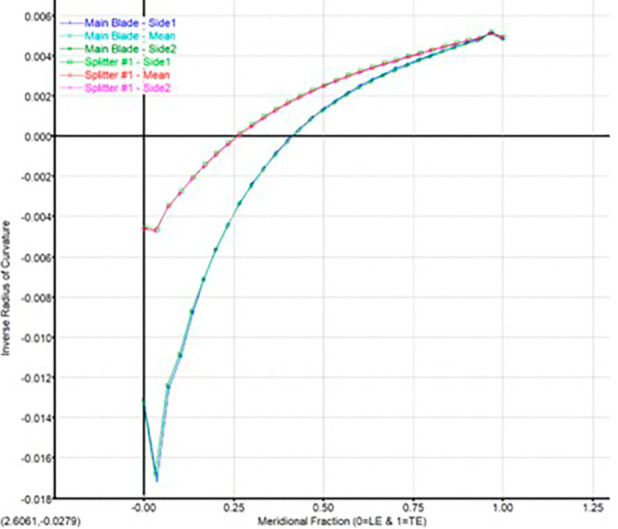
Blade-to-blade curvature graph.

**Figure 9.**
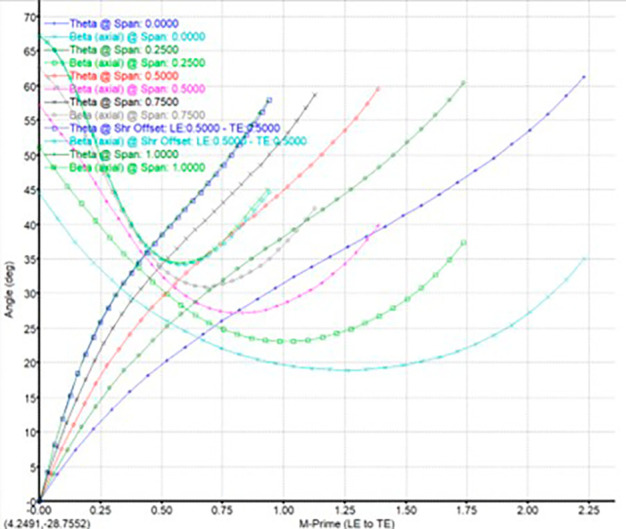
Blade angle graph.

**Figure 10.**
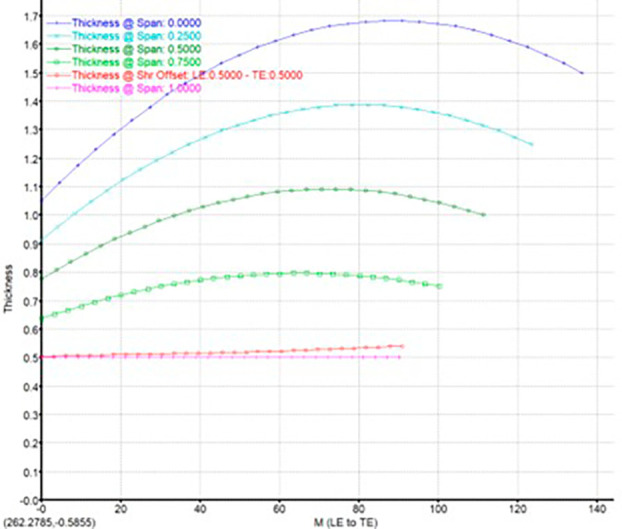
Blade thickness graph.

**Figure 11.**
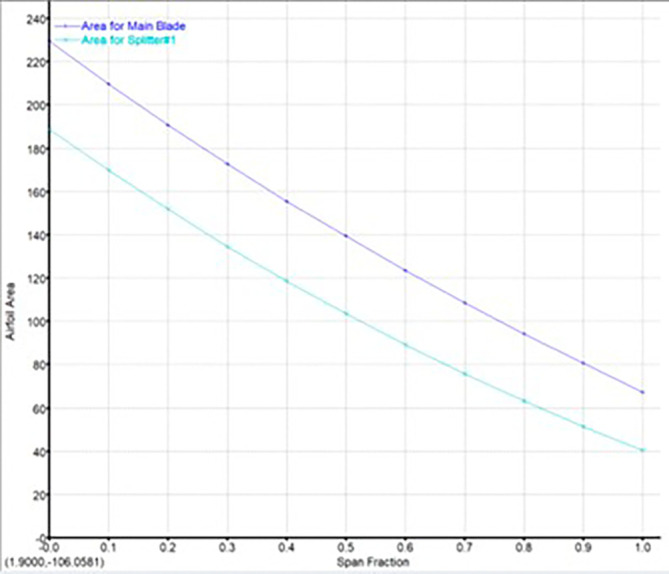
Airfoil area graph.

**Figure 12.**
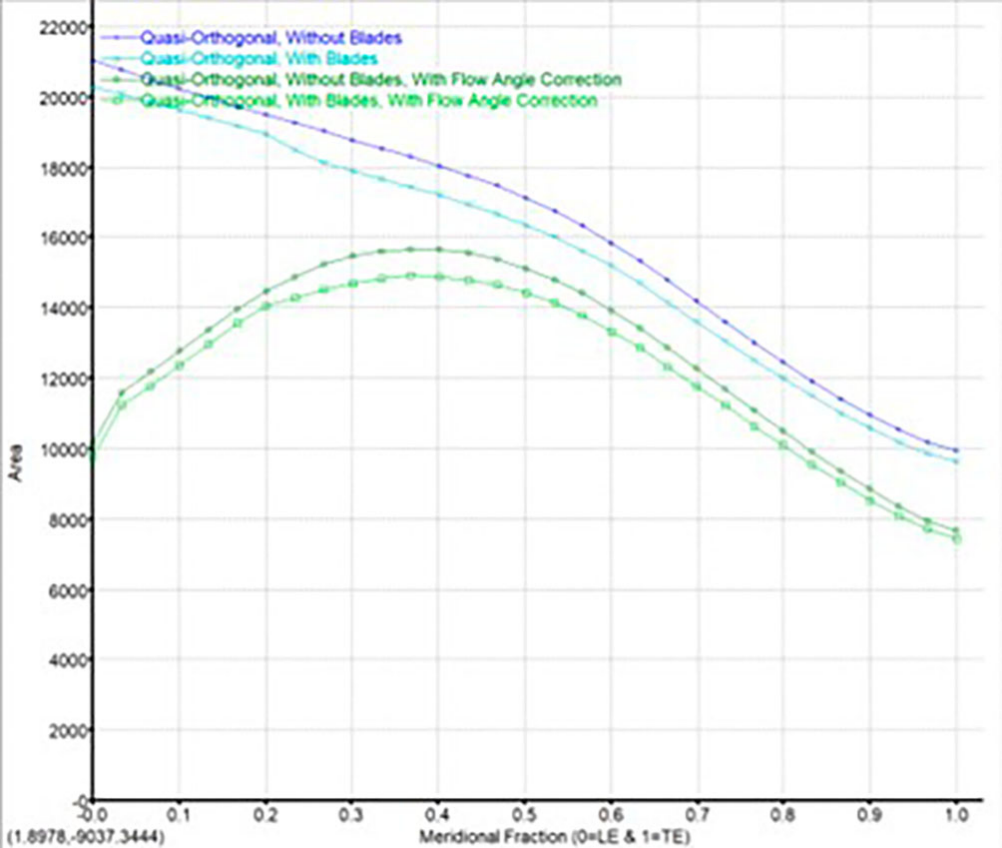
Quasi orthogonal graph.

The modeling was done using Vista CCD and BladeGen in Ansys Workbench. A 2D sketch is developed on Vista CCD based on the aerodynamic data and the geometric parameters provided. It is further edited based on changes in the Bezier curve in BladeGen. This will finally provide the 3D model as well as the blade design. Within the Vista CCD, Tables 1 and 2 serve as input sources for data entry, which undergoes processing to generate the outcomes depicted in Figure 2. Additionally, the processed data is accompanied by an illustrative impeller sketch as shown in Figure 3. Further, the data from Vista CCD is transferred to BladeGen to further model the blade based on the properties given in Figures 4 to 12. The data in these graphs are modified to obtain the required shape of the blade.

**Table 1.** Duty and aerodynamic data. ^32^

Overall pressure ratio	4.5
Mass flow	3 kg/s
Rotational speed	40000 rpm

**Table 2.** Geometric data. ^32^

Hub diameter	75 mm
Vane normal thickness	1.5 mm
Shroud diameter	180 mm
Vane normal thickness	0.5 mm
Tip clearance	0.5 mm
Main vanes	9
Inter vanes	9
Back sweep angle	45
Rake angle	30

### Meshing of centrifugal compressor

**Figures 13 and 14.**
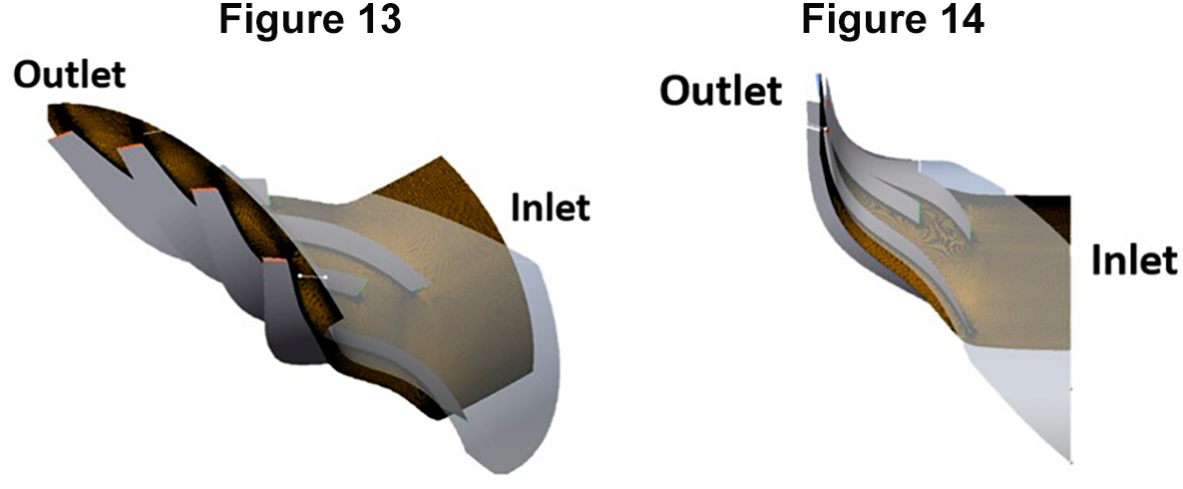
Isometric and front view of one instance of the blade respectively.

**Figures 15 and 16.**
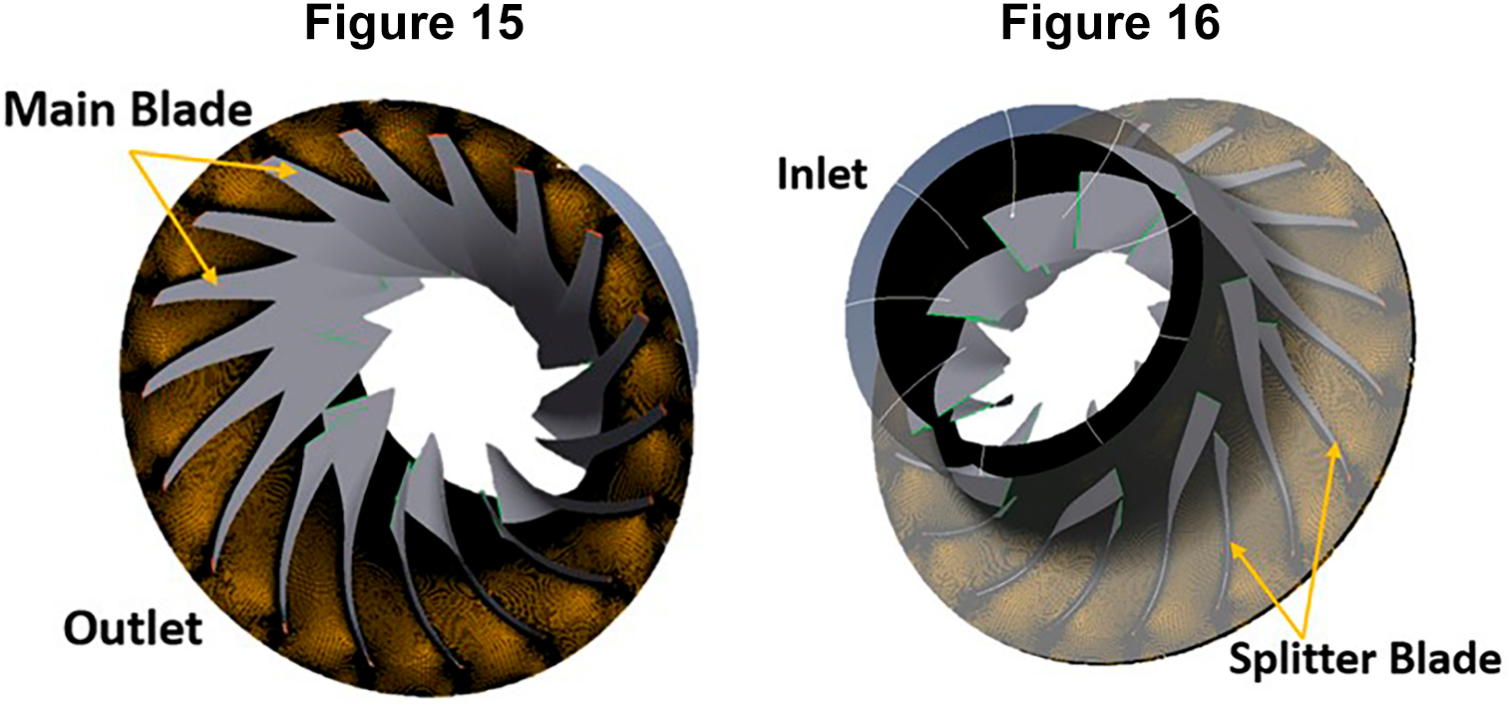
Bottom and top view of complete impeller respectively.

The meshing of the impeller geometry was performed using TurboGrid, with the blade profile from BladeGen being transferred to TurboGrid through ANSYS Workbench. The mesh details were configured according to Table 3, and a global size factor of 2 was chosen as the optimal value. This decision was based on the observation that a mesh size larger than 2 would exceed the available space within the blades. The resulting mesh consisted of a total of 1,633,660 nodes and 1,548,915 elements, as illustrated in Figures 13 to 16, showcasing each individual blade meshes and the complete 360° impeller mesh.

**Table 3.** Details of the mesh.

Method	Global size factor
Size Factor	2
Boundary Layer Refinement Control	First Element Offset Method
Cutoff Edge Split Factor Trailing	1
Target Maximum Expansion Rate	1.3
Near Wall Element Size Specification Method	Y+
Reynolds Number	1.0e6

### Boundary conditions

**Figure 17.**
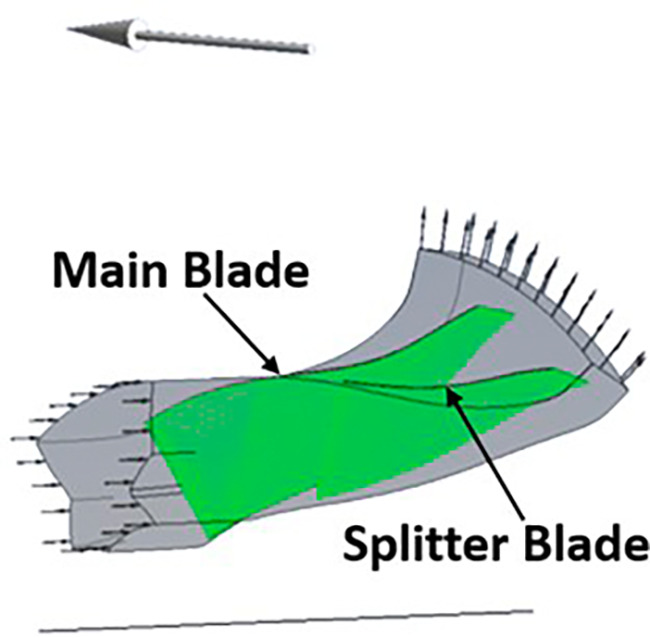
Main blade and splitter blade.

**Figure 18.**
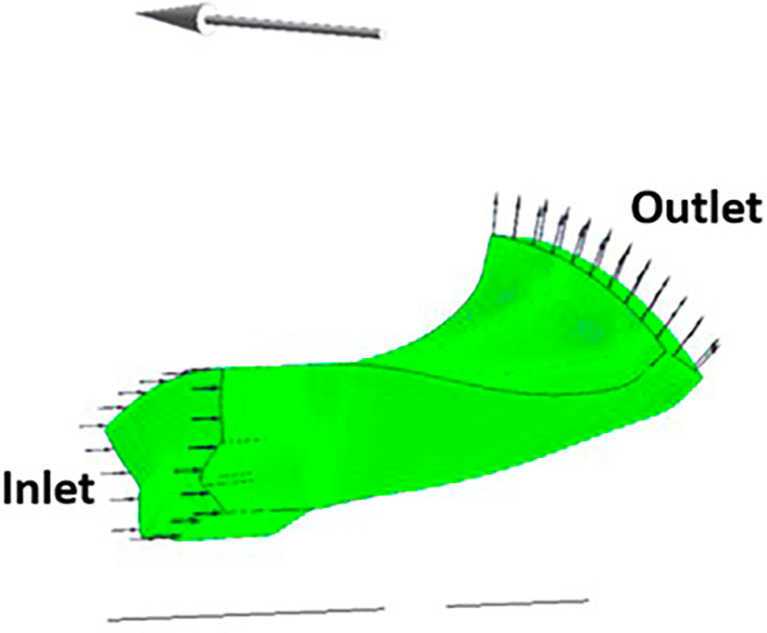
One instance of the impeller.

The boundary conditions for the centrifugal compressor were applied using the CFX Pre of ANSYS Workbench. It was provided in TurboMode. Figures 17 and 18 show the selected part for each boundary condition. No-slip wall boundary condition was given to both the splitter and the main blade. A rotational speed of 40000rpm was given in the direction of the arrow in Figure 18. Table 4 gives the data the inserted in the TurboMode that was modified for applying the required boundary conditions. The Shear Stress Transport Turbulence model in ideal gas conditions was used for this analysis.

**Table 4.** Boundary conditions for TurboMode CFX.

Machine type	Centrifugal compressor
Type	Steady State
Rotating Axis	Z
Speed	-40000
Fluid	Air Ideal Gas
Reference Pressure	0 atm
Heat Transfer	Total Energy
Turbulence	Shear Stress Transport
Inflow Pressure	101.325 kPa
Inflow Temperature	288.15 K
Static Pressure	110 kPa
Boundary Type	Wall
Wall Influence on flow	No Slip Wall

GOVERNING EQUATIONS: To solve the flow conditions, Navier-Stokes equation can be used in the following way. ^33^

$$\frac{\mathrm{\partial U}}{\mathrm{\partial t}}+\frac{\mathrm{\partial F}}{\mathrm{\partial x}}+\frac{\mathrm{\partial G}}{\mathrm{\partial y}}+\frac{\mathrm{\partial H}}{\mathrm{\partial z}}=J$$

$$U=\left\{\begin{array}{c} \rho \\ \mathrm{\rho u}\\ \mathrm{\rho v}\\ \mathrm{\rho w}\\ \rho \left(e+\frac{V^{2}}{2}\right) \end{array}\right\}$$

$$F=\left\{\begin{array}{c} \mathrm{\rho u}\\ \rho u^{2}+P-\tau_{\mathrm{xx}}\\ \mathrm{\rho vu}-\tau_{\mathrm{xy}}\\ \mathrm{\rho wu}-\tau_{\mathrm{xz}}\\ \rho \left[e+\frac{V^{2}}{2}\right]u+\mathrm{pu}-{\dot{q}}_{x}-u\tau_{\mathrm{xx}}-v\tau_{\mathrm{xy}}-w\tau_{\mathrm{xz}} \end{array}\right\}$$

$$G=\left\{\begin{array}{c} \mathrm{\rho v}\\ \mathrm{\rho uv}-\tau_{\mathrm{yx}}\\ \rho v^{2}+p-\tau_{\mathrm{yy}}\\ \mathrm{\rho wv}-\tau_{\mathrm{yz}}\\ \rho \left[e+\frac{V^{2}}{2}\right]w+\mathrm{pv}-{\dot{q}}_{y}-u\tau_{\mathrm{yx}}-v\tau_{\mathrm{yy}}-w\tau_{\mathrm{yz}} \end{array}\right\}$$

$$H=\left\{\begin{array}{c} \mathrm{\rho w}\\ \mathrm{\rho uw}-\tau_{\mathrm{zx}}\\ \mathrm{\rho vw}-\tau_{\mathrm{zy}}\\ \rho w^{2}+p-\tau_{\mathrm{zz}}\\ \rho \left[e+\frac{V^{2}}{2}\right]w+\mathrm{pw}-{\dot{q}}_{z}-u\tau_{\mathrm{zx}}-v\tau_{\mathrm{zy}}-w\tau_{\mathrm{zz}} \end{array}\right\}$$

$$J=\left\{\begin{array}{c} 0\\ \rho f_{x}\\ \rho f_{y}\\ \rho f_{z}\\ \rho \left({\mathrm{uf}}_{x}+{\mathrm{vf}}_{y}+wf_{z}\right)+\rho \dot{q} \end{array}\right\}$$

$f_{x}$ , $f_{y}$ and $f_{z}$ : Body force per unit mass in *x*, *y*, and *z* directions, respectively

$\dot{q}$ : Rate of volumetric heat addition per unit mass

${\dot{q}}_{x},{\dot{q}}_{x}\;\text{and}\;{\dot{q}}_{x}$ : Rate of volumetric heat addition per unit mass in the *x*, *y*, and *z* directions, respectively

*u*, *v*, *w*: Velocity vectors in *x*, *y*, and *z* directions, respectively

ρ : Air density (kg/m ^3^)

$\tau_{\mathrm{xx}},\tau_{\mathrm{yy}}\;\text{and}\;\tau_{\mathrm{zz}}:$ Normal stress related to time rate change of volume of the fuid element in *x*, *y*, and *z* directions, respectively.

$\tau_{\mathrm{xy}},\tau_{\mathrm{xz}},\tau_{\mathrm{yx}},\tau_{\mathrm{yz}},\tau_{\mathrm{zx},}\tau_{\mathrm{zy}}:$ Shear stress related to the time rate of change of shearing deformation of fluid element in *x*, *y*, and *z* directions, respectively.

### Grid independence check

**Figure 19.**
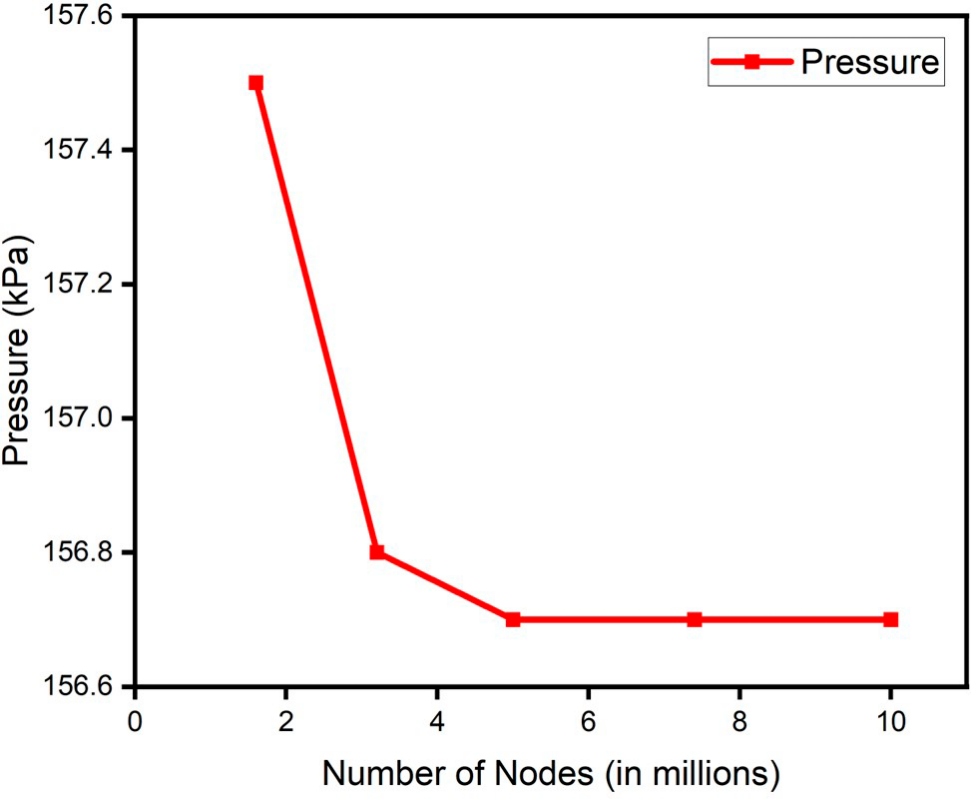
Grid independence check for pressure.

**Figure 20.**
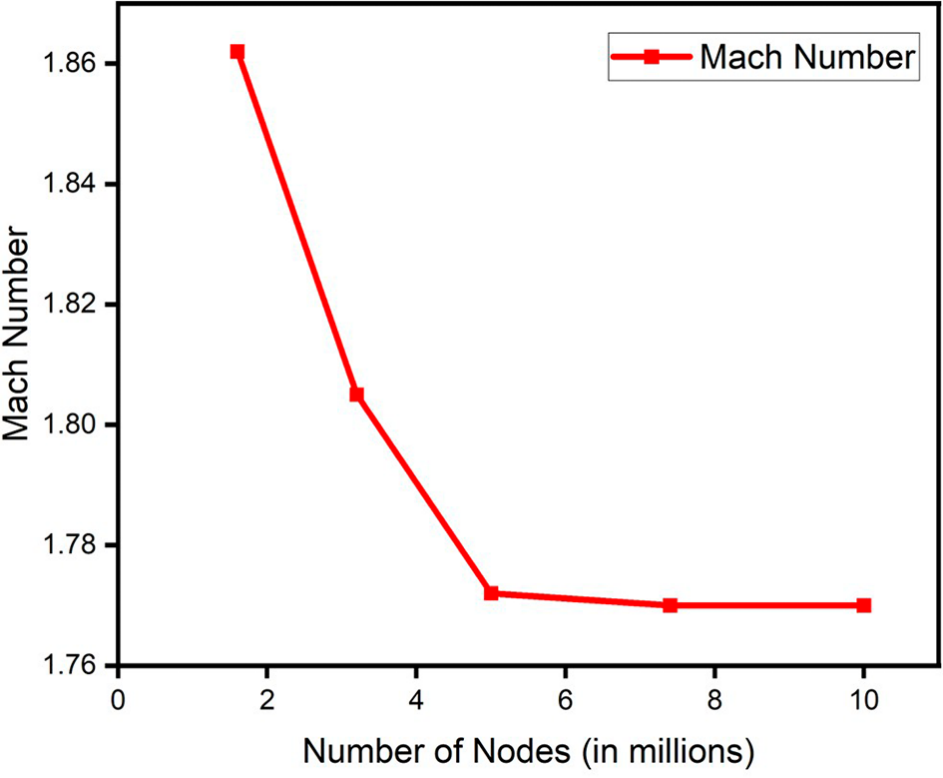
Grid independence check for mach number.

A grid independence check was conducted for all the parameters of the blade. The change in the mesh size was parameterized by the change in the global size factor. Number of mesh nodes ranging from 1.6 million to 10 million was considered. The result attained stability after 5 million nodes in most of the properties. Figures 19 and 20 show the grid independence check for pressure and Mach number respectively.

## Results and discussion

The performance study of more complicated phenomena is made possible by the CFD simulation, which creates a virtual representation of the internal flow in the centrifugal compressor. Using CFD tools, adjustments were made to the flow of a centrifugal compressor stage with all the components in situ, from the input to the output. Vector plots, contour plots, and streamline graphs are made in order to better comprehend fluid flow via the centrifugal compressor stage. The working pressure and temperature are 101.353 pa and 288.15 k respectively, with the impeller blade rotating at 40000 rpm under a 3 kg/s mass flow rate. The outcomes of this CFD analysis point to numerous possibilities for flow modeling and centrifugal compressor performance. Variations in velocity and pressure can be used to pinpoint specific locations where energy is lost. This analysis’s flow velocity pattern identifies a few significant regions where energy is lost. Results and the contour plot of the blade-to-blade view were obtained on CFX Post. The solution ran for 300 cycles.

The primary analysis was done by replicating the procedure and exact blade profiles provided in the reference. ^32^ The results obtained were within a 5% error for pressure and an 18% error for Mach Number. Table 5 gives the comparison for both pressure and Mach number values with percentage error.

**Table 5.** Comparison table for baseline analysis.

Number of nodes (in millions)	Pressure	% error wrt reference values	Mach Number	% error wrt reference values
1.6	157500	4.4400	1.862	13.270
3.2	156800	4.0178	1.805	15.920
5.0	156700	3.9566	1.772	17.466
7.4	156700	3.9566	1.770	17.559
10	156700	3.9566	1.770	17.559
Based on the reference paper ^32^	150500		2.147	

**Figure 21.**
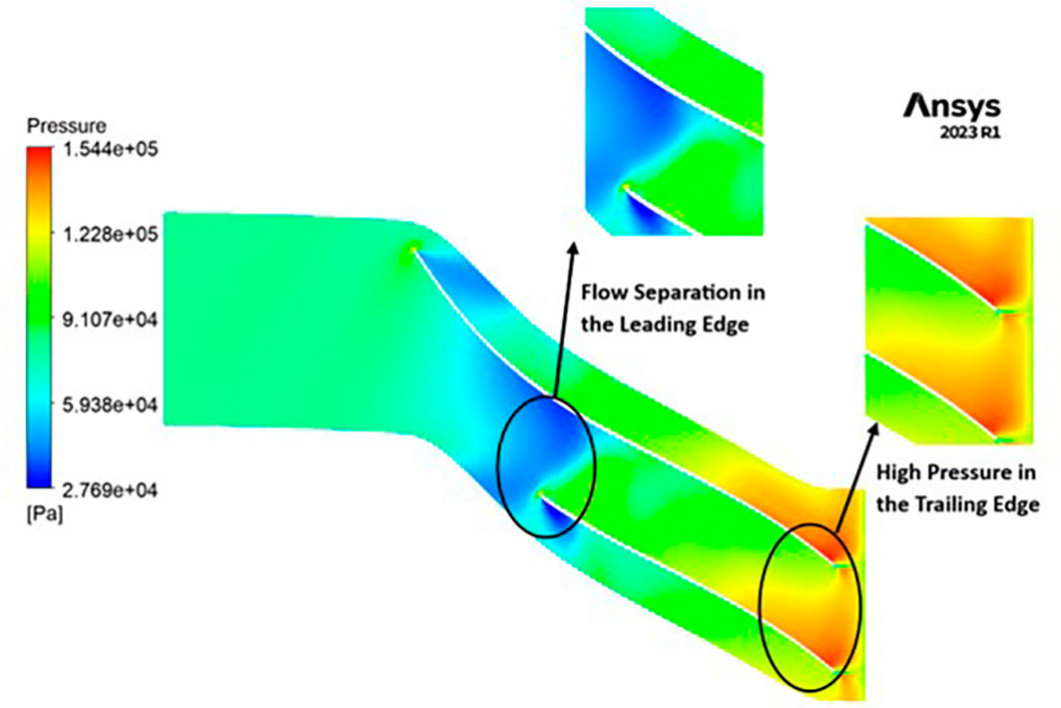
Pressure flow contour for baseline analysis.

PRESSURE: The pressure variations encountered by the impeller are notable, spanning from 27,690 to 154,400 Pascals. Notably, the leading edge faces the lowest pressure levels, exhibiting distinct flow separations in this region. Across the blade span, a moderate pressure of 91,070 Pascals is consistently observed. Moving towards the trailing edge, the impeller experiences elevated pressure ranging from 122,800 to 154,400 Pascals, as illustrated in Figure 21. Additionally, it is evident that high-pressure zones are forming at the tip of each blade.

**Figure 22.**
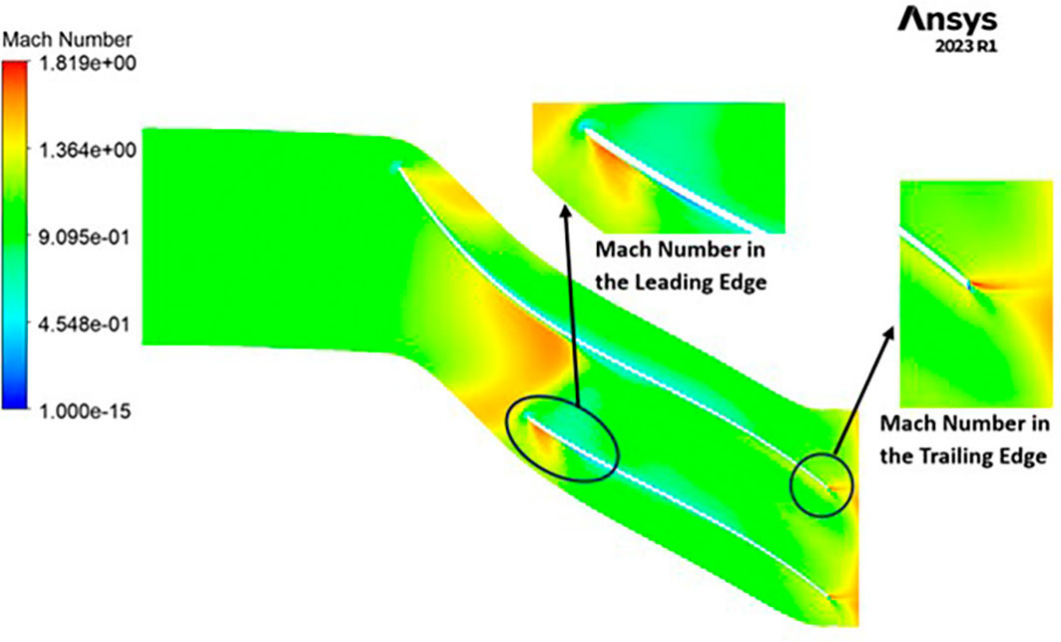
Mach number flow contour for baseline analysis.

MACH NUMBER: The Mach number exhibits a wide range, spanning from near-zero values up to 1.819. Notably, a distinct high Mach number is prominently observed at the leading edge of the impeller. A closer examination of Figure 22 reveals that in proximity to the blade, the Mach number diminishes significantly, approaching nearly zero. Across the entirety of the impeller’s span, the Mach number maintains an approximate value of 0.9. As one progresses towards the trailing edge of the impeller, high Mach numbers resurface. Additionally, there is a subtle presence of lower Mach numbers at the blade tips.

**Figure 23.**
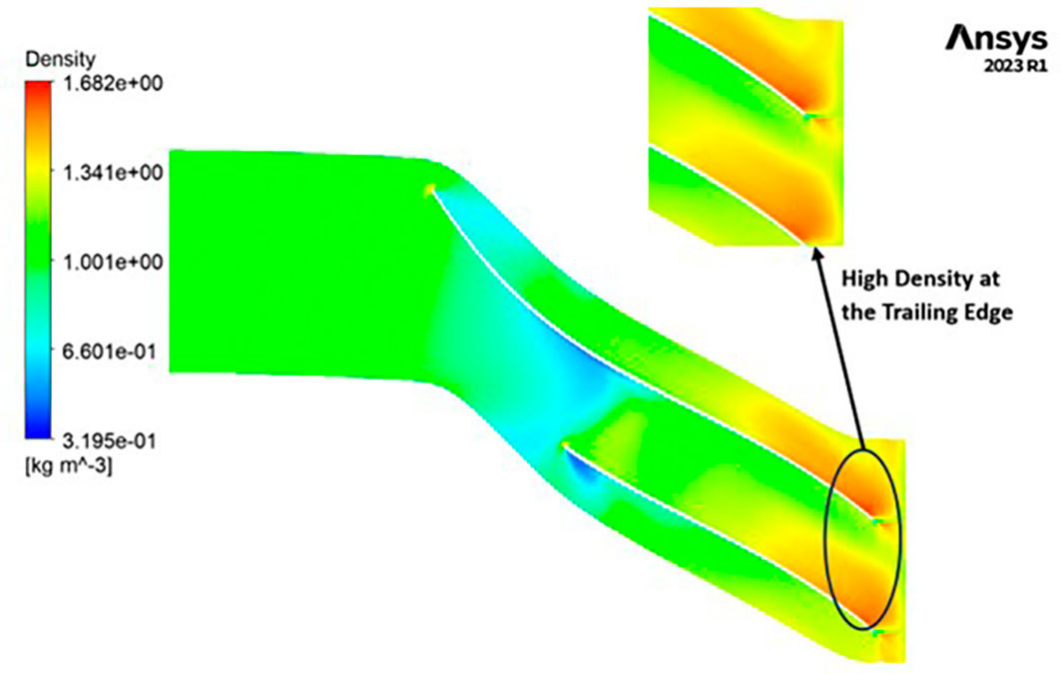
Density flow contour for baseline analysis.

DENSITY: The density distribution within the impeller displays a notable range, spanning from 0.32 to 1.682 kilograms per cubic meter (kg/m ^3^). At the leading edge of the impeller, there is a discernible low-density region, with values ranging from approximately 0.3 to 0.6 kg/m ^3^. Across the entirety of the impeller’s span, the density is generally within the range of 1 to 1.3 kg/m ^3^. Conversely, at the trailing edge, a region of high density, measuring around 1.6 kg/m ^3^, is evident. Upon closer examination of Figure 23, it becomes apparent that high-density regions are observed at the blade tips on the leading edge, while at the blade tips on the trailing edge, a density of approximately 1 kg/m ^3^ is observed.

**Figure 24.**
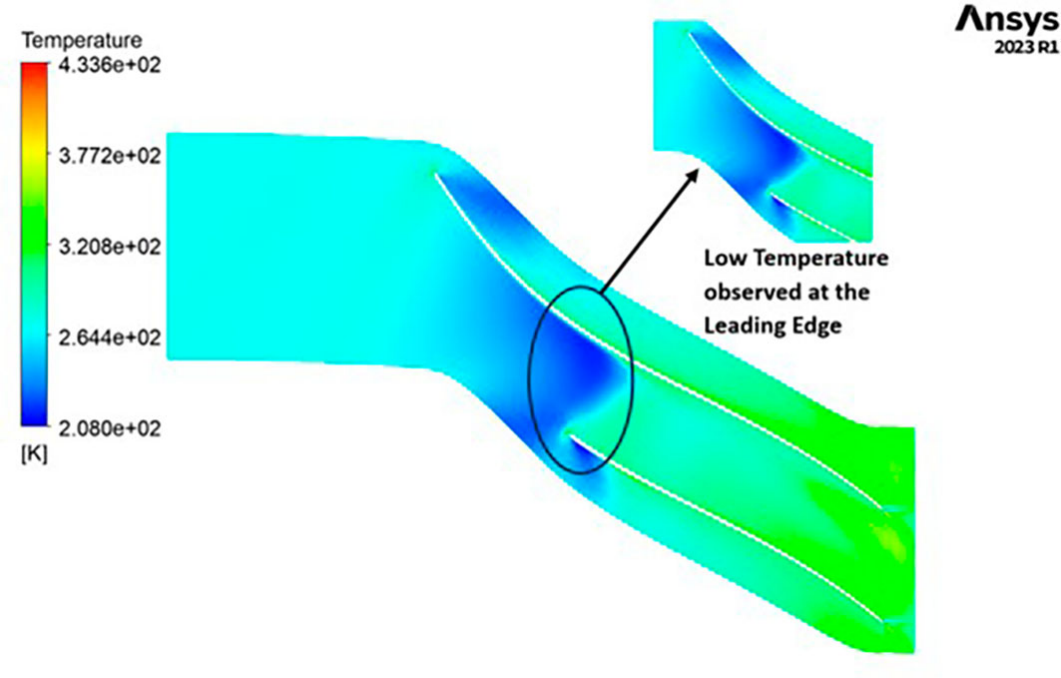
Temperature flow contour for baseline analysis.

TEMPERATURE: The temperature distribution within the impeller exhibits a range between 208 Kelvin (K) and 433 K. Notably, conspicuous contours depicting lower temperatures are evident at the leading edge, as depicted in Figure 24. Across the entire span of the impeller, temperatures remain relatively consistent, falling within the range of 264 K to 320 K. Towards the trailing edge, a subtle tendency towards higher temperatures, approximately around 370 K, becomes apparent. A closer examination reveals faint indications of elevated temperatures reaching 433 K at the blade tips.

**Figure 25.**
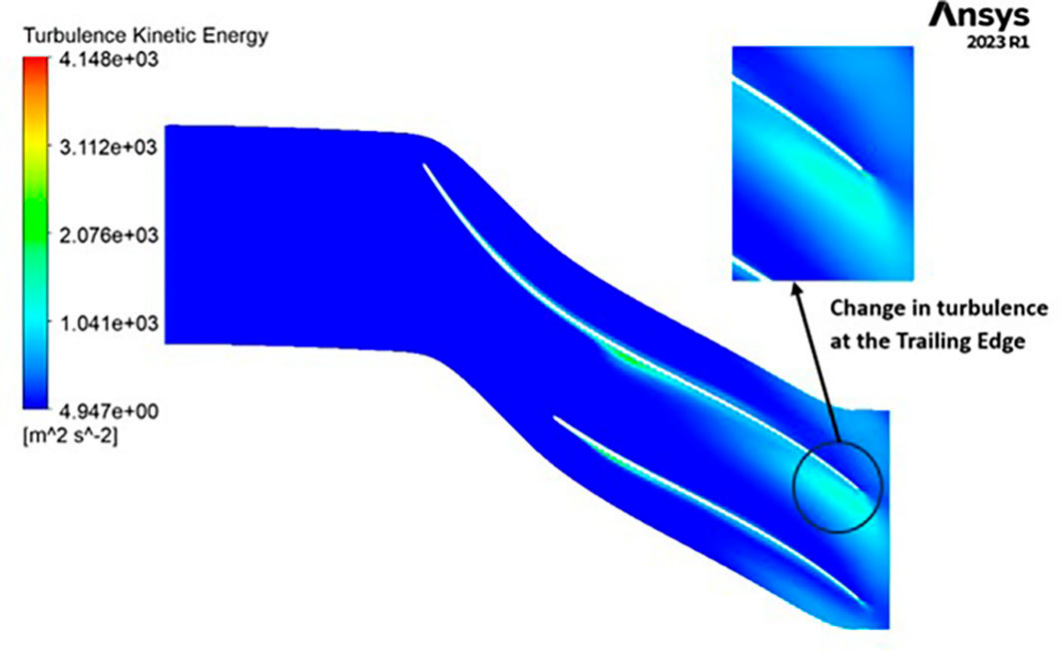
Turbulence flow contour for baseline analysis.

TURBULENCE: The observed turbulence kinetic energy spans a range from 4.9 to 4148 square meters per second squared (m ^2^/s ^2^). Along the entirety of the blade’s span, a consistent turbulence level of 1041 m ^2^/s ^2^ is evident from Figure 25. Furthermore, the turbulence maintains a relatively constant and low value, specifically at 4.947 m ^2^/s ^2^ throughout. Notably, a predominant concentration of turbulence is observed at the trailing edge of the impeller.

**Figure 26.**
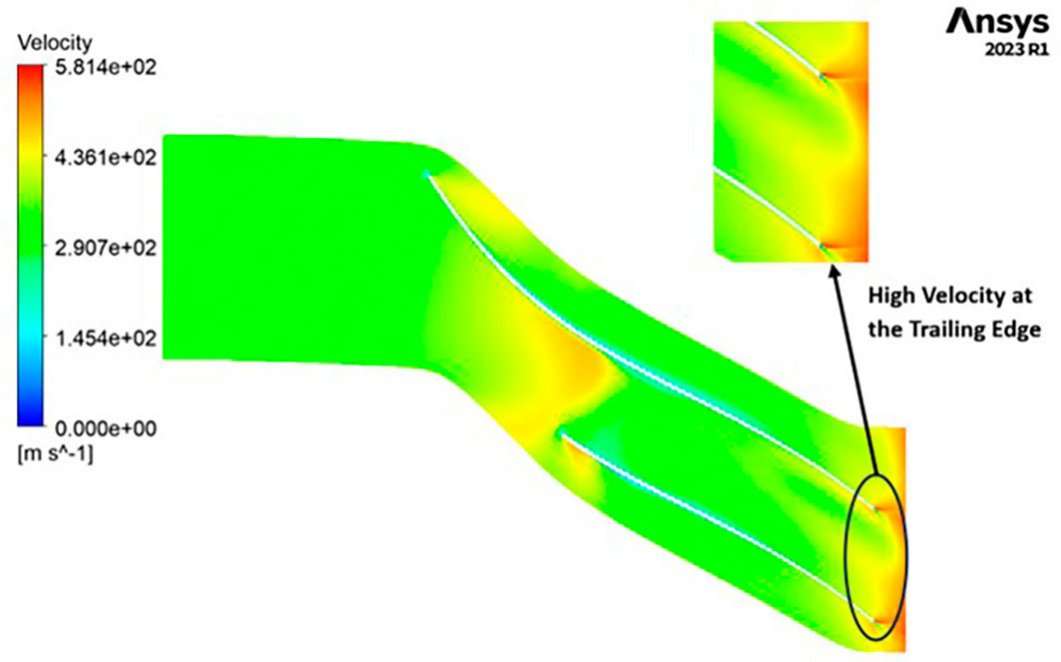
Velocity flow contour for baseline analysis.

VELOCITY: The velocity distribution within the impeller reveals a range from 0 to 581.4 meters per second (m/s). Notably, distinctive contours depicting high velocity, specifically at 581.4 m/s, are prominently observed at the trailing edge. Across the entire span of the impeller, the velocity consistently falls within the range of 290 to 430 m/s. Upon closer scrutiny of Figure 26, it becomes evident that the velocity along the blade exhibits significantly lower values in comparison to other regions within the impeller.

## Optimized blade analysis

An alternative turbulence model was employed in the aforementioned analysis to discern its efficacy and alignment with values reported in the reference paper. The transition to the k-Epsilon turbulence model in Ansys CFX was executed by modifying the turbulence model within the Turbo mode. The Shear Stress Transport (SST) turbulence model is a hybrid approach that combines elements of the k-ε and k-ω models, excelling in accuracy for complex flows with adverse pressure gradients and separation. It is particularly favoured for turbomachinery simulations involving intricate boundary layers. In contrast, the k- Epsilon turbulence model is computationally more efficient but tends to provide less accurate results, making it suitable for simpler flow scenarios with fewer complexities. The results of pressure and Mach number compared to the SST model and the reference paper model are shown in Tables 6 and 7 with the percentage increment. From the results in k-Epsilon turbulence model, an increment in the pressure compared to the SST model is observed. Furthermore, a slight decrease in the Mach number is also observed.

**Table 6.** Comparison table for baseline analysis of SST turbulence model.

Number of nodes (in millions)	Pressure SST	% error wrt reference values	Mach Number SST	% error wrt reference values
1.6	157500	4.4400	1.862	13.270
3.2	156800	4.0178	1.805	15.920
5.0	156700	3.9566	1.772	17.466
7.4	156700	3.9566	1.770	17.559
10	156700	3.9566	1.770	17.559
Based on the reference paper ^32^	150500		2.147	

**Table 7.** Comparison table for baseline analysis of k-Epsilon turbulence model.

Number of nodes (in millions)	Pressure k-epsilon	% error wrt reference values	Mach Number k-epsilon	% error wrt reference values
1.6	160000	6.312	1.784	16.91
3.2	160400	6.578	1.737	19.09
5.0	160600	6.711	1.728	19.51
7.4	160600	6.711	1.728	19.51
10	160600	6.711	1.728	19.51
Based on the reference paper ^32^	150500		2.147	

**Figure 27.**
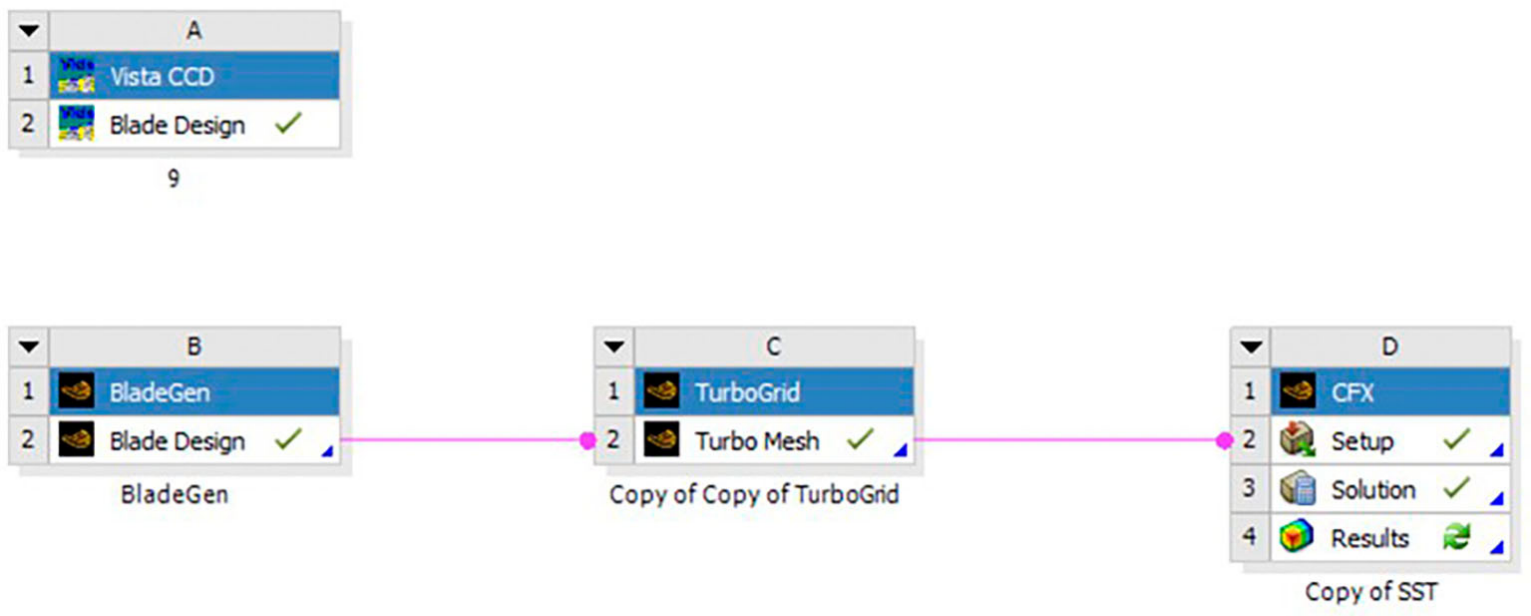
Project schematics for optimization in each mesh size.

**Figure 28.**
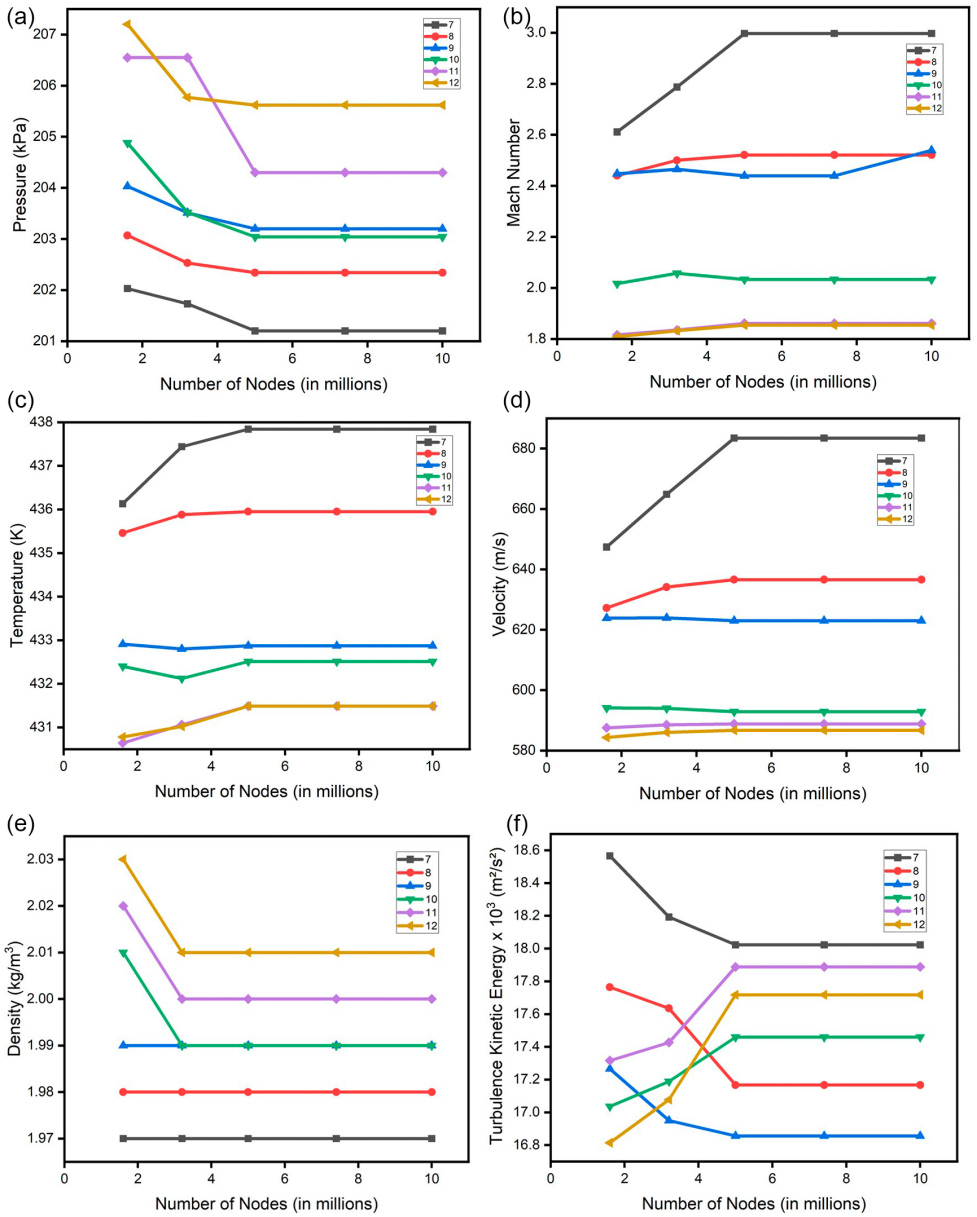
Grid independence check for SST turbulence (a) Pressure; (b) Mach number; (c) Temperature; (d) Velocity; (e) Density and (f) Turbulence kinetic energy.

**Figure 29.**
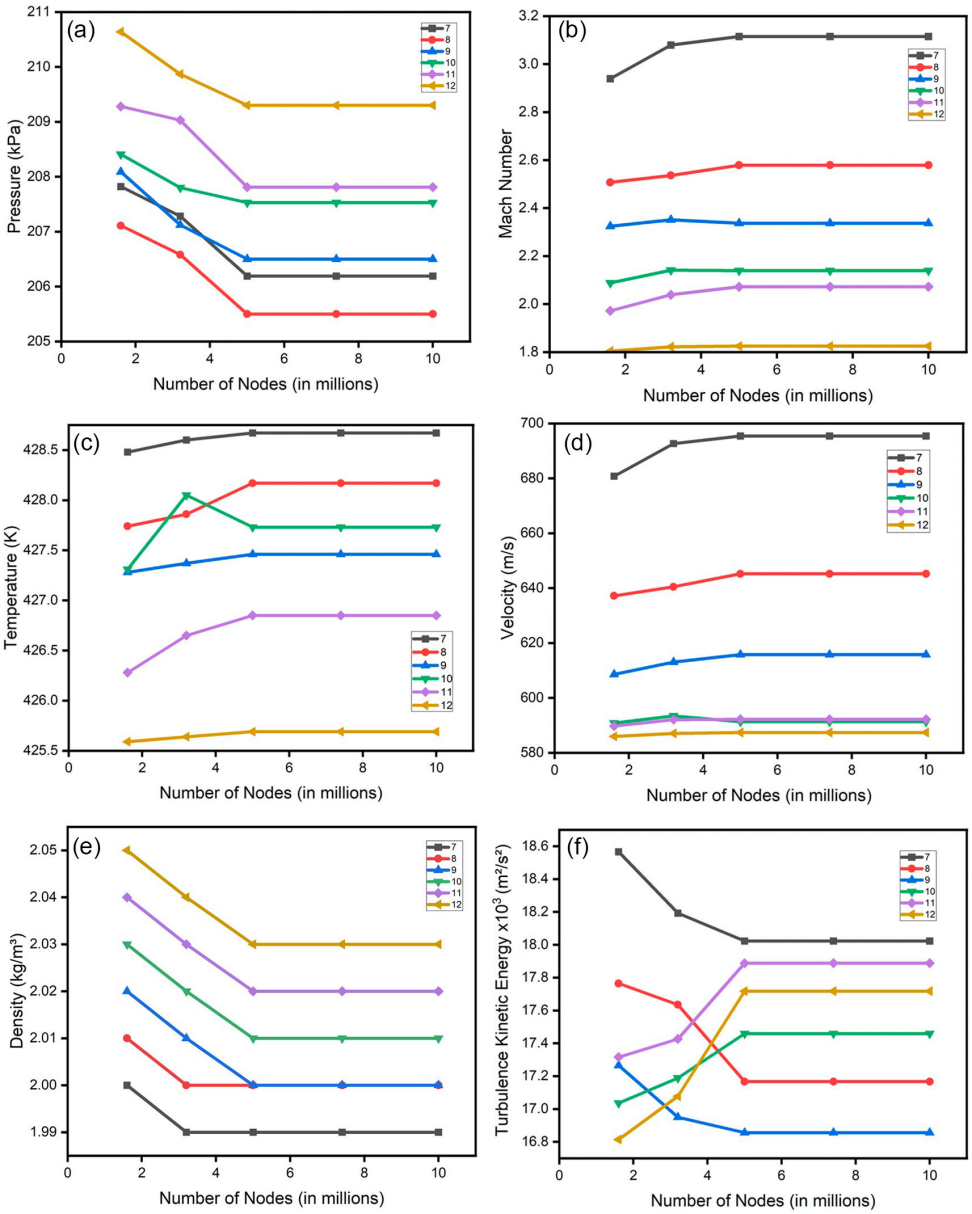
Grid independence check for k-Epsilon turbulence (a) Pressure; (b) Mach number; (c) Temperature; (d) Velocity; (e) Density and (f) Turbulence kinetic energy.

A minor adjustment was introduced to the blade geometry curve, resulting in improved outcomes for both turbulence models. Consequently, this refined design was employed to investigate optimization based on variations in the number of blades and hub diameter as shown in Table 8. The aforementioned analyses using both turbulence models were reiterated across five additional scenarios, involving an increase in the number of blades coupled with a simultaneous reduction in hub diameter. Figure 27 illustrates the project schematics for one mesh size, with an analogous creation of four additional mesh sizes to facilitate a comprehensive assessment of grid independence. For each case, a grid independence check was done in both SST turbulence and k-Epsilon turbulence. This check was done for pressure, Mach number, density, velocity, turbulence, and temperature as shown in Figures 28 and 29. All the obtained results of the SST turbulence are shown in Table 9 and that of k-Epsilon is shown in Table 10.

**Table 8.** Geometrical changes for optimization study.

Blade number	Hub diameter
7	85
8	80
9	75
10	70
11	65
12	60

**Table 9.** Results table for all cases in SST turbulence.

Number of nodes (in million)	7	8	9	10	11	12
Pressure (kPa)						
1.6	202.03	203.07	204.03	204.88	206.55	207.20
3.2	201.73	202.53	203.51	203.52	206.55	205.77
5.0	201.20	202.34	203.20	203.04	204.30	205.62
7.4	201.20	202.34	203.20	203.04	204.30	205.62
10	201.20	202.34	203.20	203.04	204.30	205.62
Mach Number						
1.6	2.611	2.440	2.447	2.017	1.816	1.809
3.2	2.787	2.500	2.465	2.057	1.835	1.832
5.0	2.997	2.521	2.439	2.033	1.861	1.854
7.4	2.997	2.521	2.439	2.033	1.861	1.854
10	2.997	2.521	2.539	2.033	1.861	1.854
Temperature (K)						
1.6	436.13	435.46	432.91	432.40	430.64	430.78
3.2	437.44	435.88	432.80	432.12	431.06	431.02
5.0	437.84	435.95	432.87	432.51	431.49	431.49
7.4	437.84	435.95	432.87	432.51	431.49	431.49
10	437.84	435.95	432.87	432.51	431.49	431.49
Velocity (ms ^−1^)						
1.6	647.39	627.21	623.89	594.11	587.53	584.32
3.2	664.85	634.14	623.96	593.99	588.54	586.03
5.0	683.46	636.58	622.99	592.89	588.85	586.72
7.4	683.46	636.58	622.99	592.89	588.85	586.72
10	683.46	636.58	622.99	592.89	588.85	586.72
Density (kg m ^−3^)						
1.6	1.97	1.98	1.99	2.01	2.02	2.03
3.2	1.97	1.98	1.99	1.99	2.00	2.01
5.0	1.97	1.98	1.99	1.99	2.00	2.01
7.4	1.97	1.98	1.99	1.99	2.00	2.01
10	1.97	1.98	1.99	1.99	2.00	2.01
Turbulence Kinetic Energy (m ^2^s ^−2^)						
1.6	18565.6	17764.5	17265.2	17036.4	17315.6	16814.1
3.2	18192.3	17634.8	16949.4	17188.7	17426.6	17077.6
5.0	18022.6	17167.0	16855.6	17459.1	17887.8	17717.5
7.4	18022.6	17167.0	16855.6	17459.1	17887.8	17717.5
10	18022.6	17167.0	16855.6	17459.1	17887.8	17717.5

**Table 10.** Results table for all cases in k-Epsilon Turbulence.

Number of nodes (in million)	7	8	9	10	11	12
Pressure (kPa)						
1.6	207.82	207.11	208.09	208.41	209.28	210.64
3.2	207.28	206.58	207.12	207.80	209.03	209.87
5.0	206.19	205.50	206.50	207.53	207.81	209.30
7.4	206.19	205.50	206.50	207.53	207.81	209.30
10	206.19	205.50	206.50	207.53	207.81	209.30
Mach Number						
1.6	2.939	2.507	2.324	2.088	1.972	1.804
3.2	3.079	2.536	2.351	2.141	2.039	1.822
5.0	3.115	2.579	2.337	2.140	2.072	1.825
7.4	3.115	2.579	2.337	2.140	2.072	1.825
10	3.115	2.579	2.337	2.140	2.072	1.825
Temperature (K)						
1.6	428.48	427.74	427.28	427.31	426.28	425.59
3.2	428.60	427.86	427.37	428.05	426.65	425.64
5.0	428.67	428.17	427.46	427.73	426.85	425.69
7.4	428.67	428.17	427.46	427.73	426.85	425.69
10	428.67	428.17	427.46	427.73	426.85	425.69
Velocity (ms ^−1^)						
1.6	680.79	637.18	608.57	590.85	589.76	585.95
3.2	692.65	640.49	613.04	593.46	592.08	587.07
5.0	695.41	645.23	615.79	591.33	592.23	587.39
7.4	695.41	645.23	615.79	591.33	592.23	587.39
10	695.41	645.23	615.79	591.33	592.23	587.39
Density (kg m ^−3^)						
1.6	2.00	2.01	2.02	2.03	2.04	2.05
3.2	1.99	2.00	2.01	2.02	2.03	2.04
5.0	1.99	2.00	2.00	2.01	2.02	2.03
7.4	1.99	2.00	2.00	2.01	2.02	2.03
10	1.99	2.00	2.00	2.01	2.02	2.03
Turbulence Kinetic Energy (m ^2^s ^−2^)						
1.6	14180.0	14194.0	14727.0	12816.8	12577.9	12413.7
3.2	13986.8	14960.2	15422.9	12680.8	12571.7	12420.5
5.0	13979.4	14313.5	15923.8	13731.3	13528.7	13234.5
7.4	13979.4	14313.5	15923.8	13731.3	13528.7	13234.5
10	13979.4	14313.5	15923.8	13731.3	13528.7	13234.5

**Figure 30.**
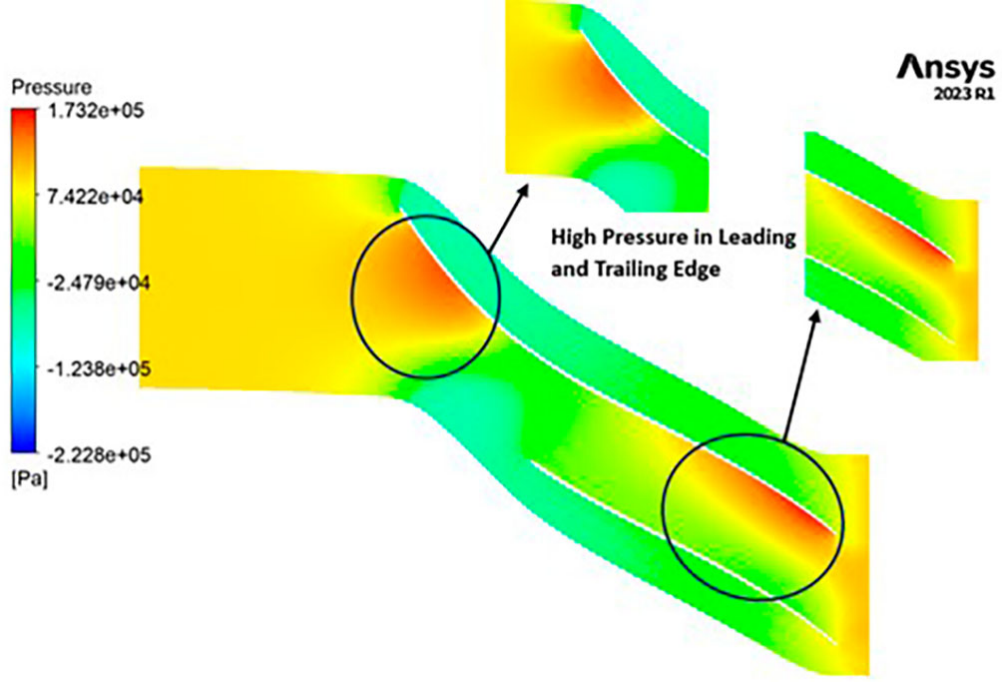
Pressure flow contour for baseline analysis (k-Epsilon).

**Figure 31.**
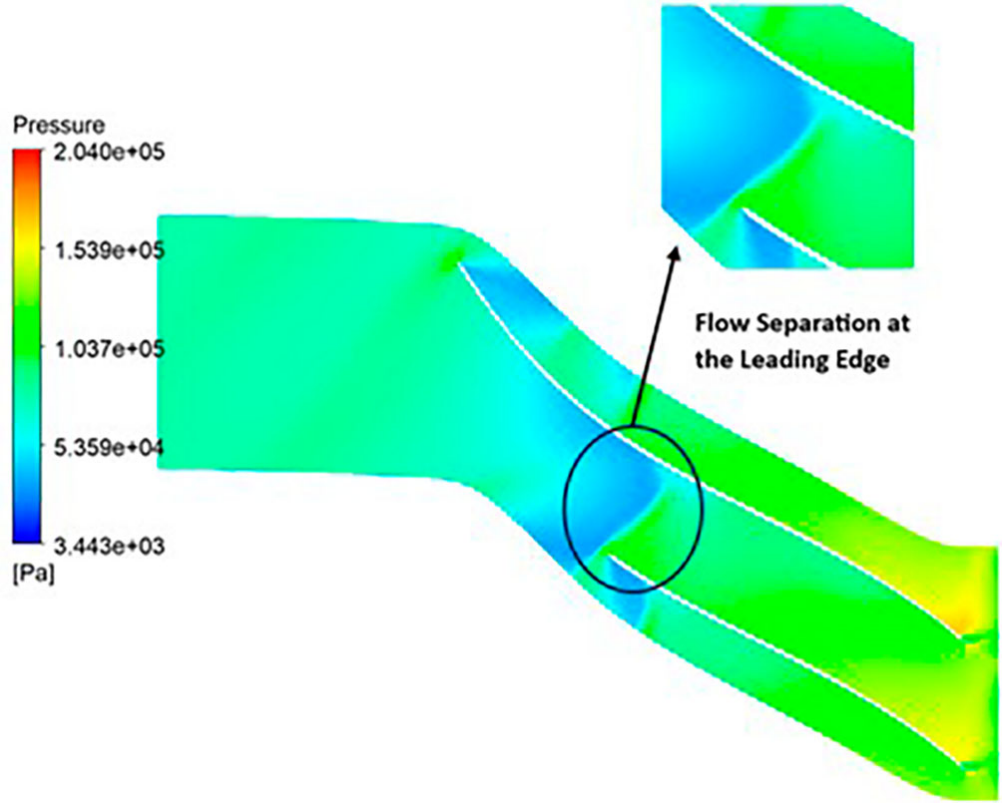
Pressure flow contour for the modified blade (SST).

**Figure 32.**
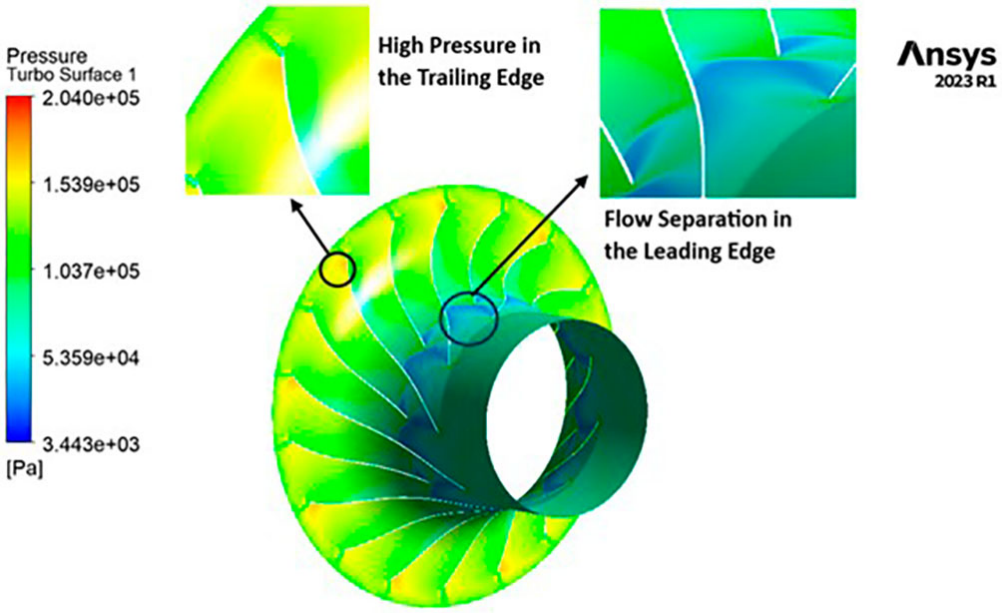
3D view pressure flow contour.

**Figure 33.**
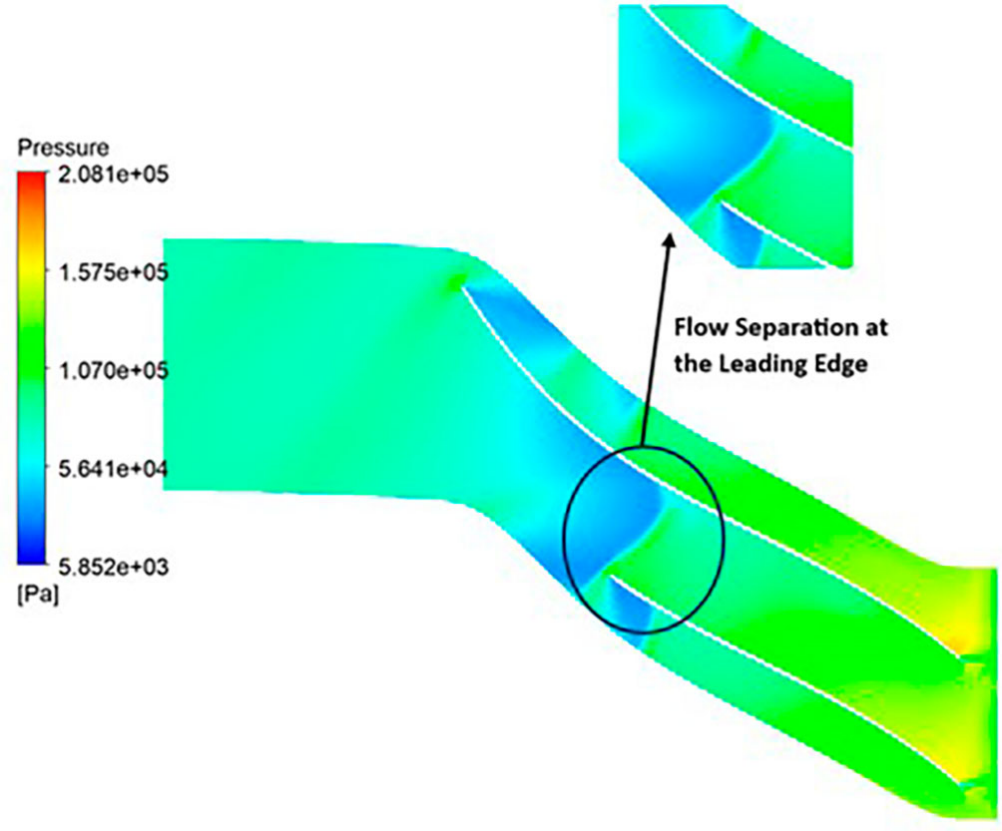
Pressure flow contour for the modified blade (k-Epsilon).

**Figure 34.**
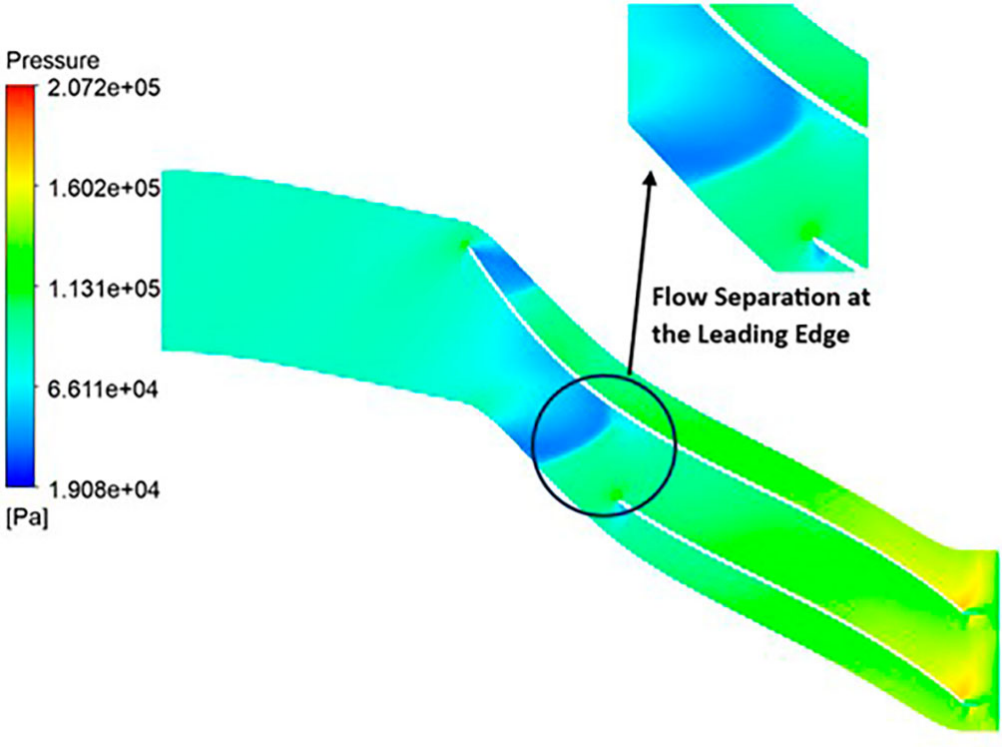
Pressure flow contour for the optimized blade (SST).

**Figure 35.**
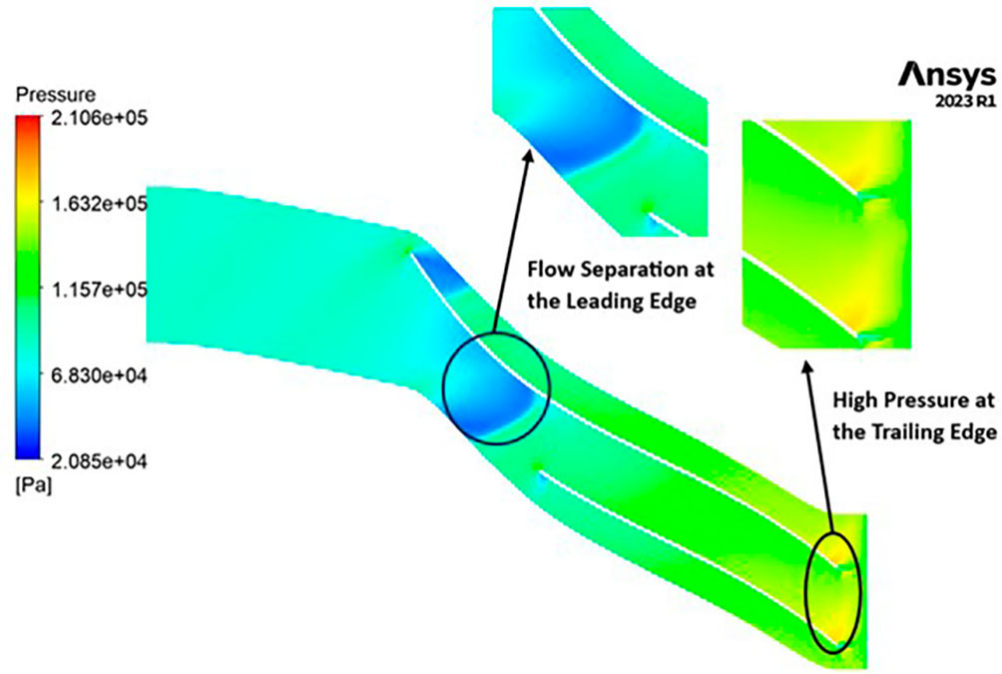
Pressure flow contour for the optimized blade (k-Epsilon).

PRESSURE: The analysis outcomes underscore the substantial improvements achieved by implementing the optimized blade design, resulting in enhanced performance metrics compared to the original configuration. Notably, the optimized blades demonstrate a significant increase in pressure ratio, indicative of improved fluid compression capabilities. In the baseline analysis, the maximum impeller pressure recorded in the SST model stands at 154.4 kPa, as depicted in Figure 21, while in the k-Epsilon model, it reaches 173.2 kPa. Noteworthy variations in flow contours are observed between these two cases. In the SST model, flow separation and low-pressure regions are notable at the leading edge, whereas the k-Epsilon model exhibits high-pressure regions both at the leading and trailing edges, as illustrated in Figure 30. Upon introducing slight modifications to the blade design, the contours align more closely with those seen in Figure 21. However, there is an increase in pressure profile, reaching 204.05 kPa in the SST model ( Figures 31 and 32) and 208.15 kPa in the k-Epsilon turbulence model ( Figure 33). Furthermore, a consistent trend of pressure increase is observed with variations in blade number and hub diameter. Consequently, the optimized configuration is attained with 12 blades and a hub diameter of 60 mm. Both the SST and k-Epsilon turbulence models exhibit distinct flow separation at the leading edge, as evidenced in Figures 34 and 35, respectively.

**Figure 36.**
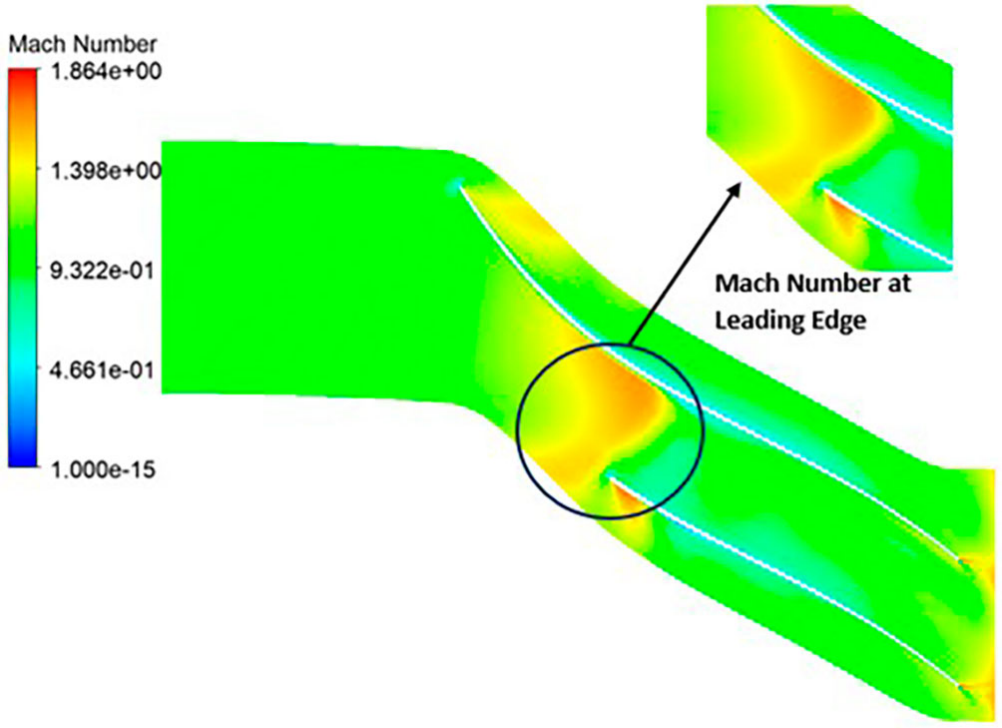
Mach Number contour for baseline analysis (k-Epsilon).

**Figure 37.**
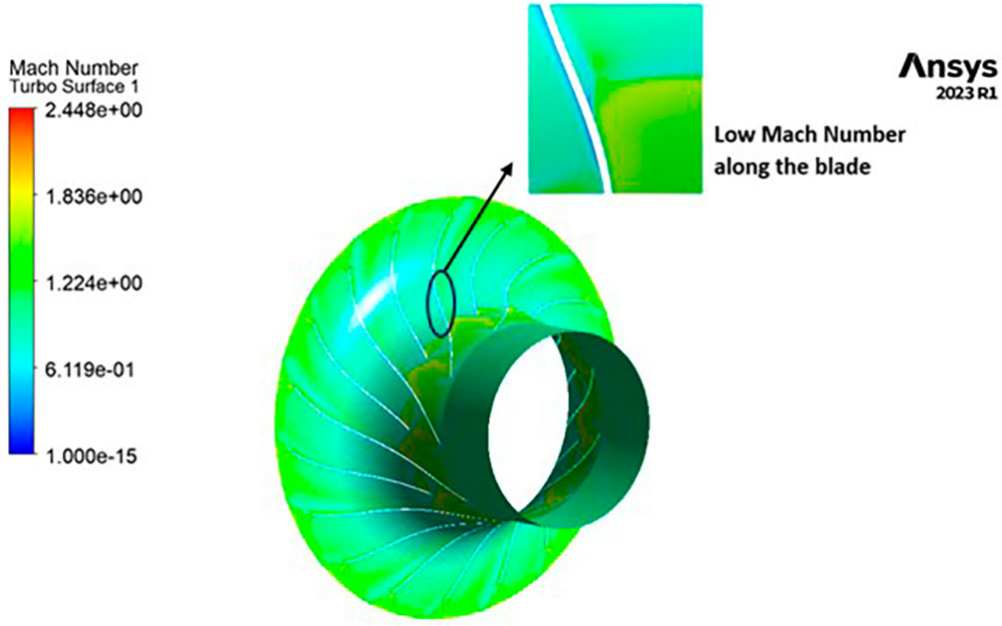
3D view Mach Number flow contour.

**Figure 38.**
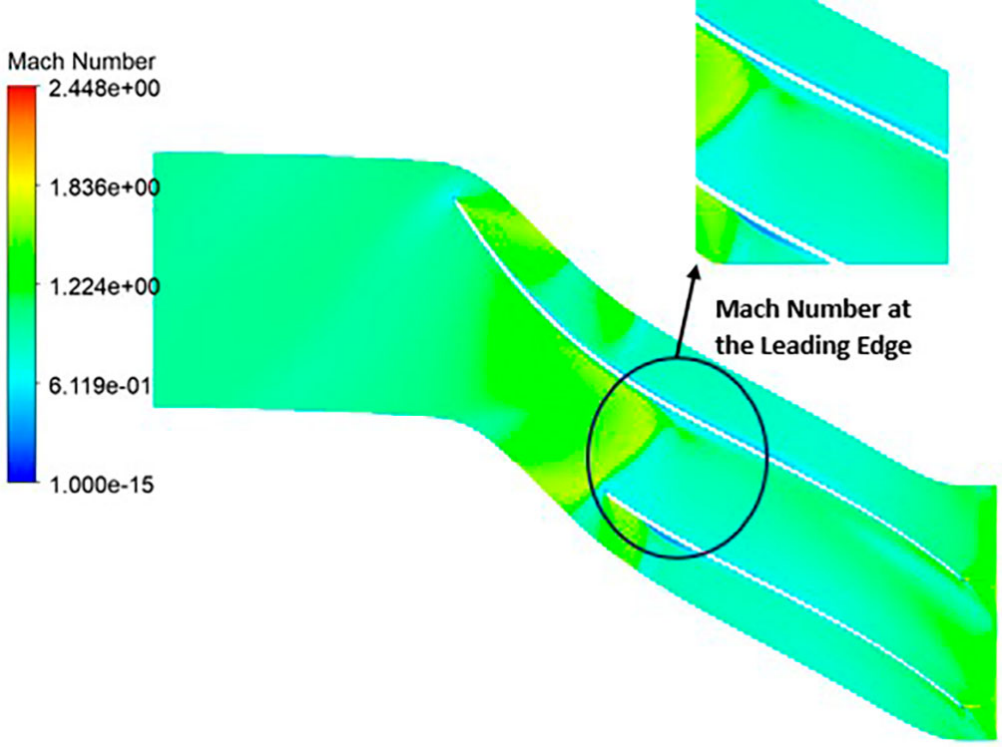
Mach Number contour for the modified blade (SST).

**Figure 39.**
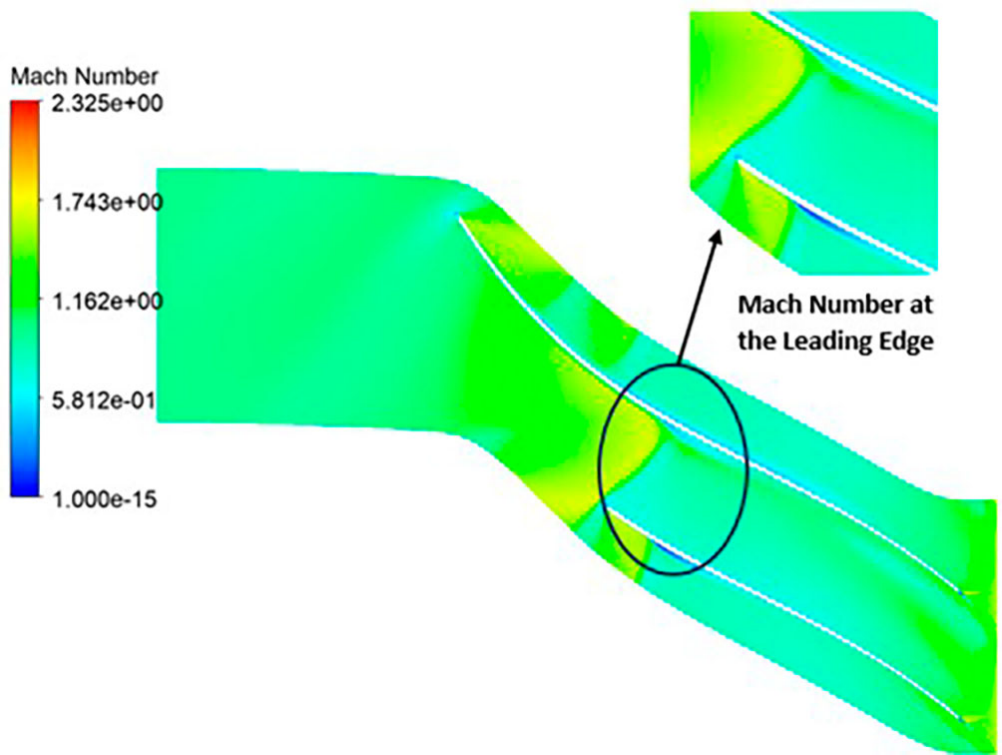
Mach Number contour for the modified blade (k-Epsilon).

**Figure 40.**
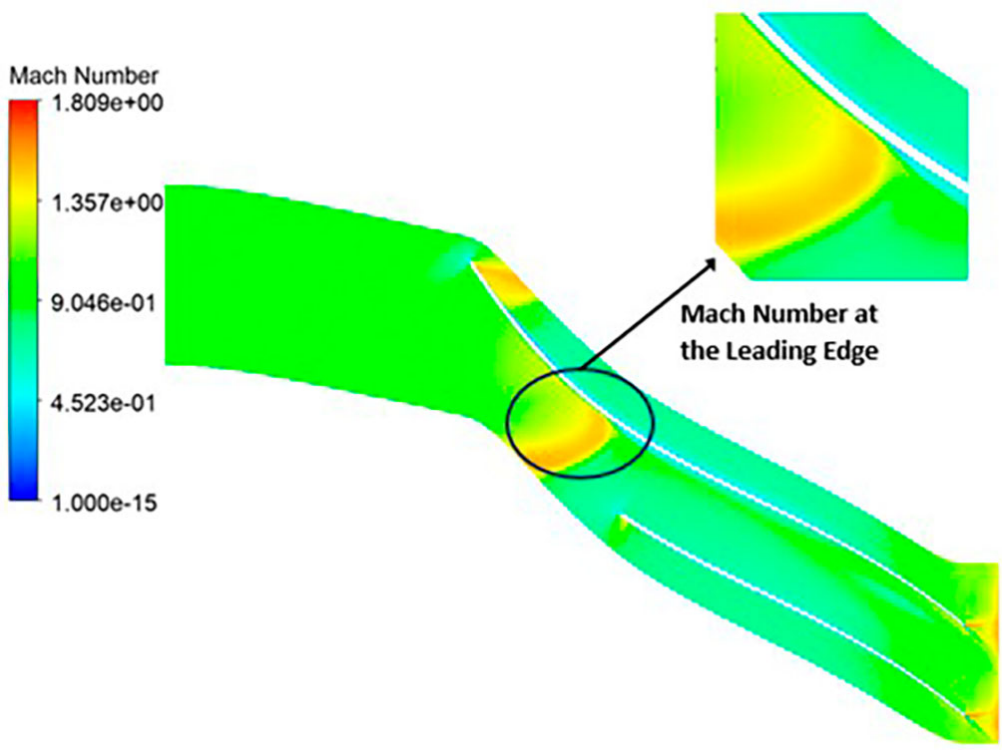
Mach Number contour for the optimized blade (SST).

**Figure 41.**
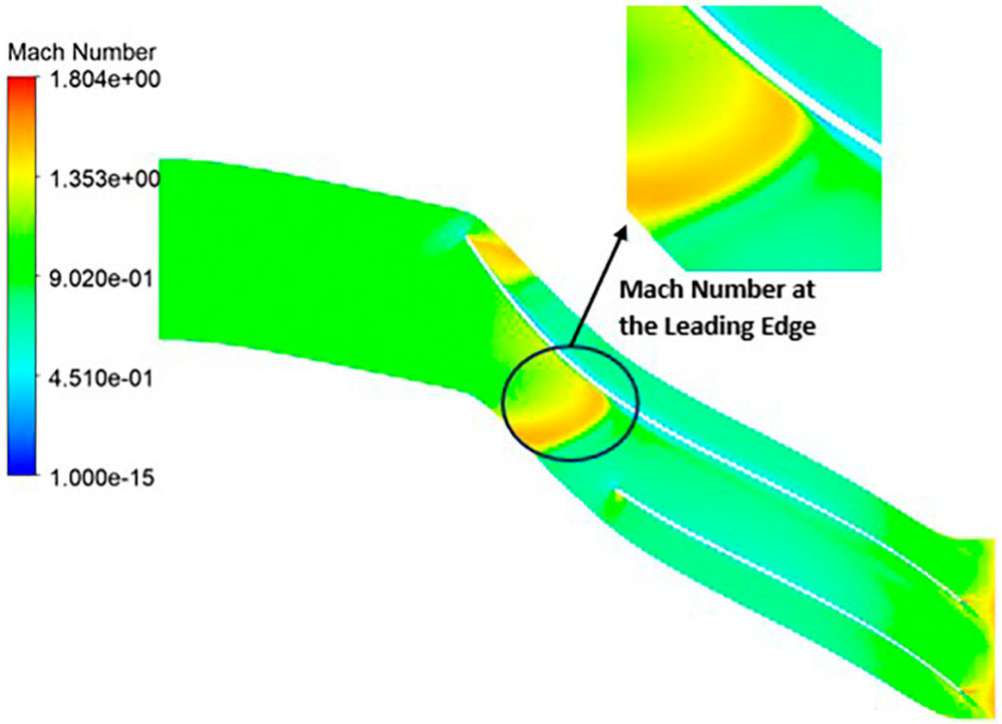
Mach number contour for the optimized blade (k-Epsilon).

MACH NUMBER: The analysis results underscore that the implementation of the optimized blade design brings about significant enhancements in the Mach number, representing a more controlled and less turbulent flow regime compared to the original configuration. This characteristic offers distinct advantages by reducing the risk of aerodynamic losses and mitigating flow separation phenomena. In the context of the k-Epsilon turbulence model, the Mach number contours exhibit relative stability, with only a slight increase in Mach number evident in Figure 36. However, with the modified blade design, a notable surge in the Mach number to 2.4 is observed in Figures 37, 38, and 39. It is noteworthy that while the maximum Mach number increases, the contours reflect a shift towards lower Mach numbers at both the leading and trailing edges. High Mach numbers are primarily confined to the blade tips within the impeller. Furthermore, a detailed examination of Tables 9 and 10 reveals a clear trend: a lower number of blades and a higher hub diameter tend to elevate the Mach number. Conversely, as the blade count increases and the hub diameter decreases, the Mach number decreases, resulting in reduced turbulence levels. Consequently, the optimized Mach number is achieved with 12 blades and a 60mm hub diameter, as illustrated in Figures 40 and 41.

**Figure 42.**
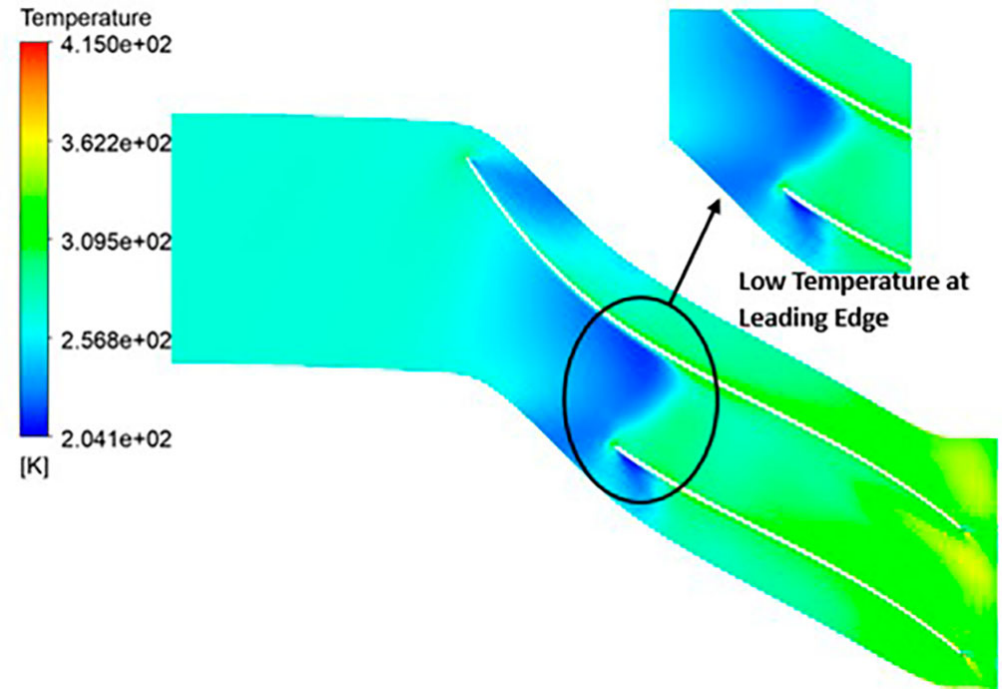
Temperature flow contour for baseline analysis (k-Epsilon).

**Figure 43.**
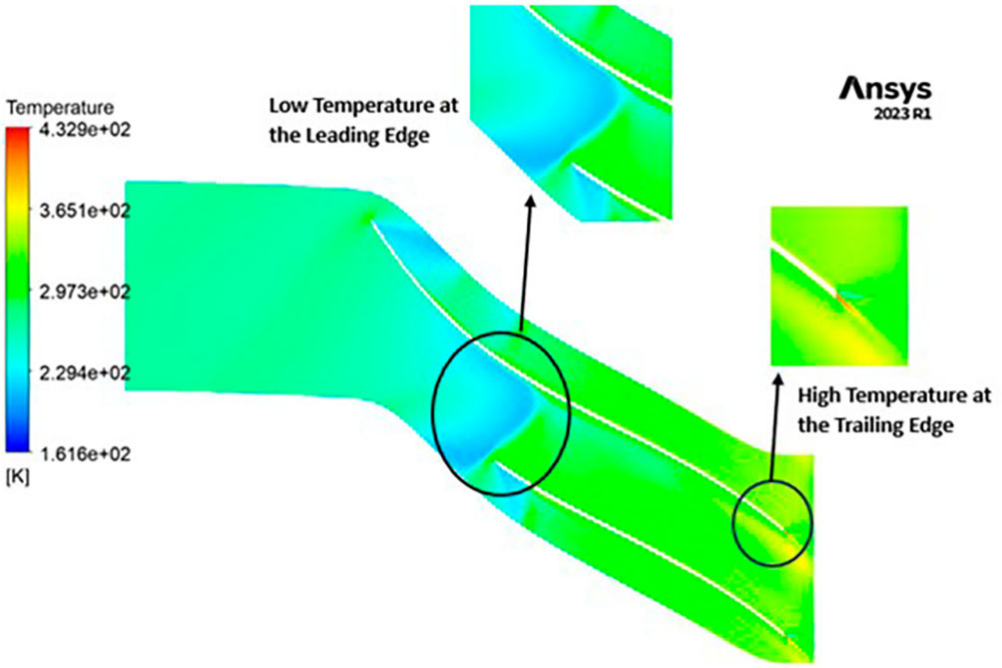
Temperature flow contour for the modified blade (SST).

**Figure 44.**
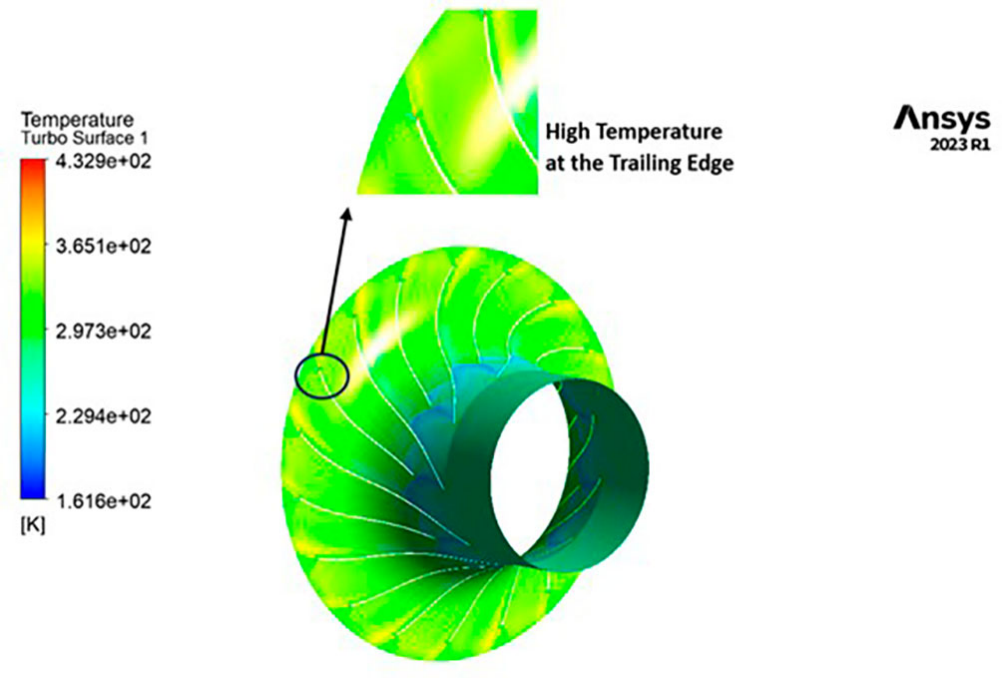
3D view temperature flow contour.

**Figure 45.**
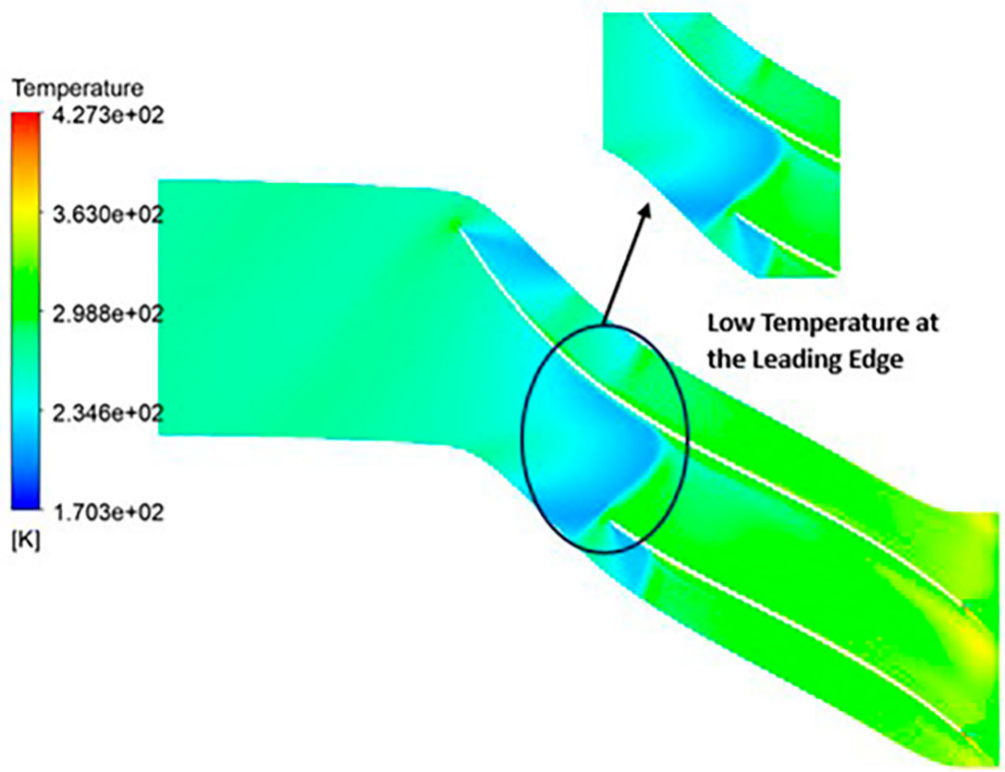
Temperature flow contour for the modified blade (k-Epsilon).

**Figure 46.**
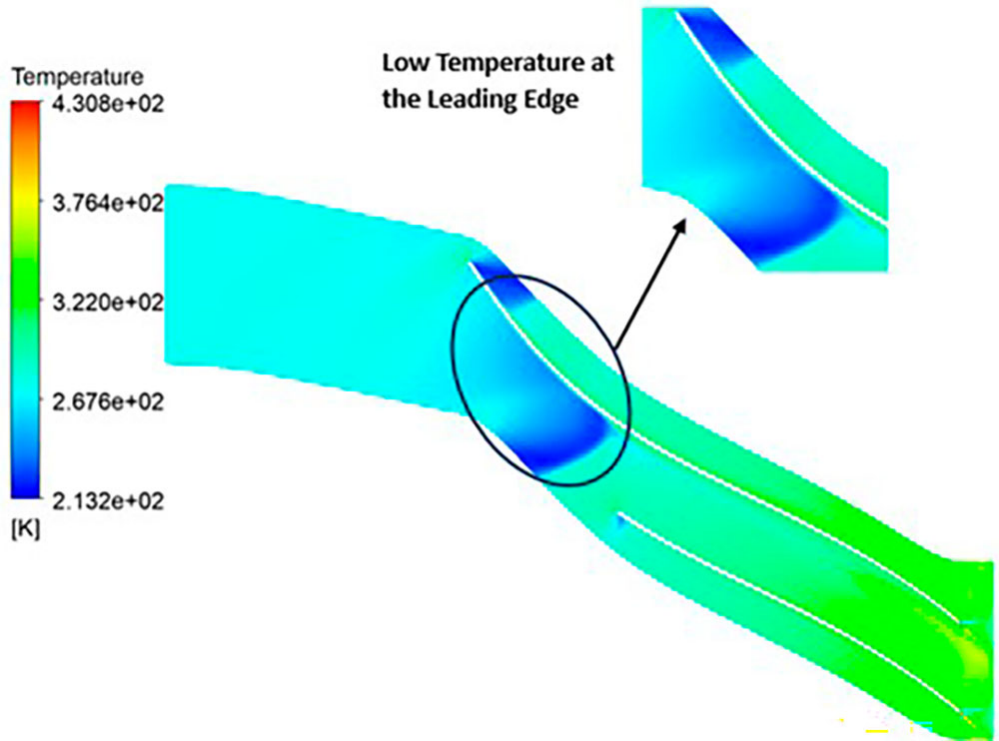
Temperature flow contour for the optimized blade (SST).

**Figure 47.**
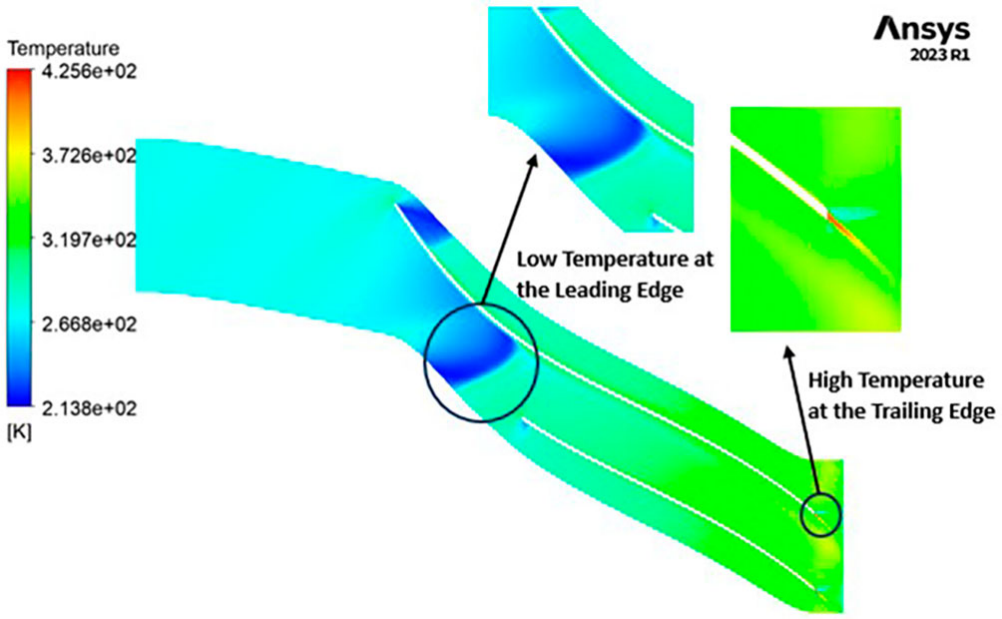
Temperature flow contour for the optimized blade (k-Epsilon).

TEMPERATURE: The analysis results substantiate that the implementation of the optimized blade design yields significant enhancements in temperature performance compared to the original configuration. Notably, the optimized blade design leads to an increase in temperature within the compressor. This outcome is a direct consequence of the improved energy transfer from the optimized blades to the fluid, resulting in a noticeable temperature depletion. It is crucial to emphasize that this decrease in temperature remains well within acceptable operational limits. Remarkably, evident contours depicting lower temperatures are observed prominently at the leading edge, as depicted in Figures 42 to 45. Across the entirety of the impeller span, temperatures remain relatively uniform. Toward the trailing edge, there is a subtle trend toward higher temperatures. A closer examination reveals faint indications of elevated temperatures at the blade tips. The lowest temperature range is achieved in the case of 12 blades for both turbulence models, as illustrated in Figures 46 and 47.

**Figure 48.**
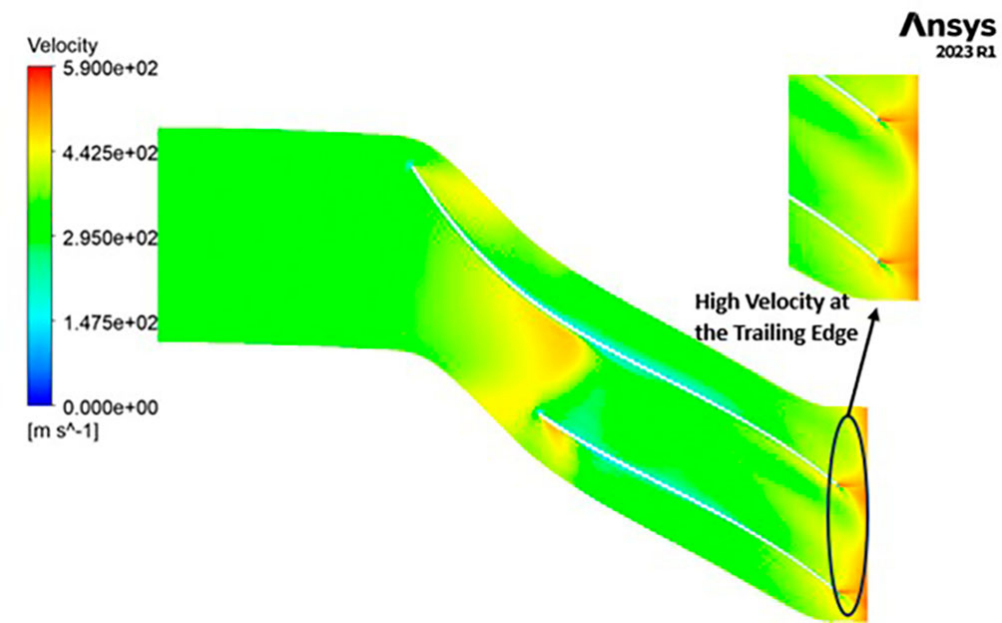
Velocity flow contour for baseline analysis (k-Epsilon).

**Figure 49.**
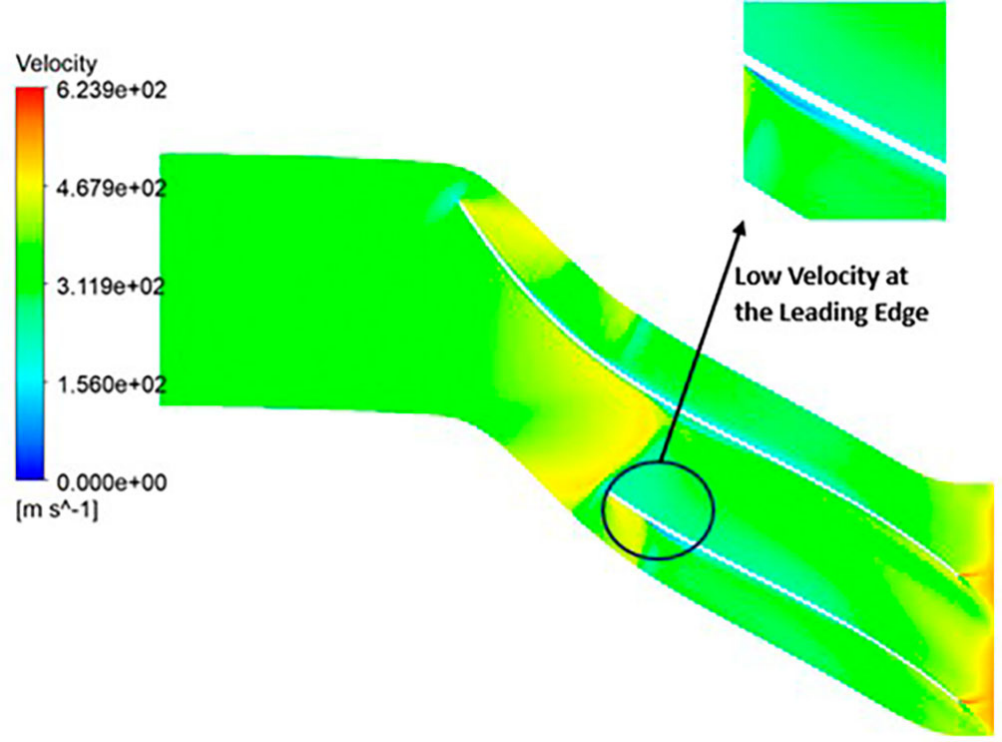
Velocity flow contour for the modified blade (SST).

**Figure 50.**
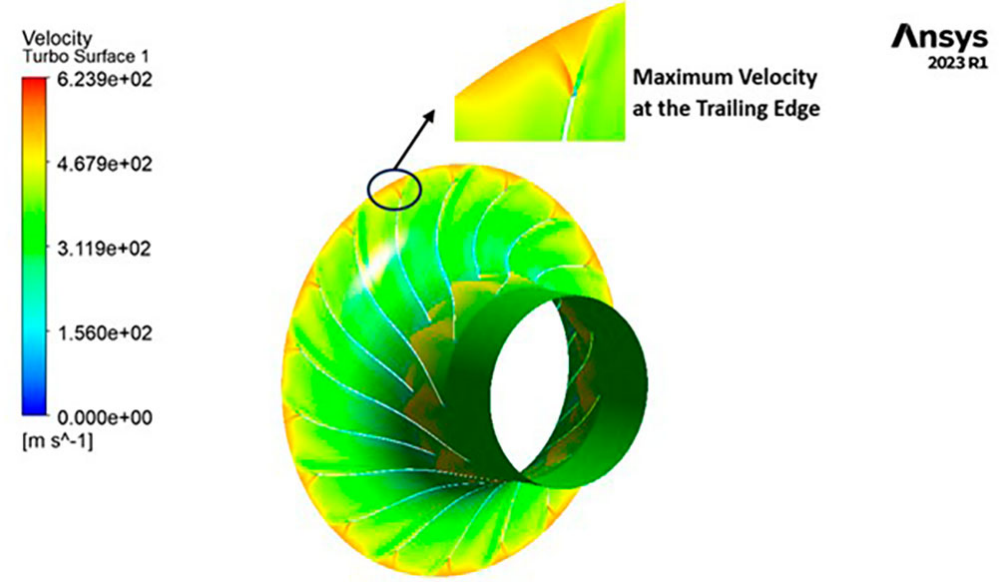
3D view velocity flow contour.

**Figure 51.**
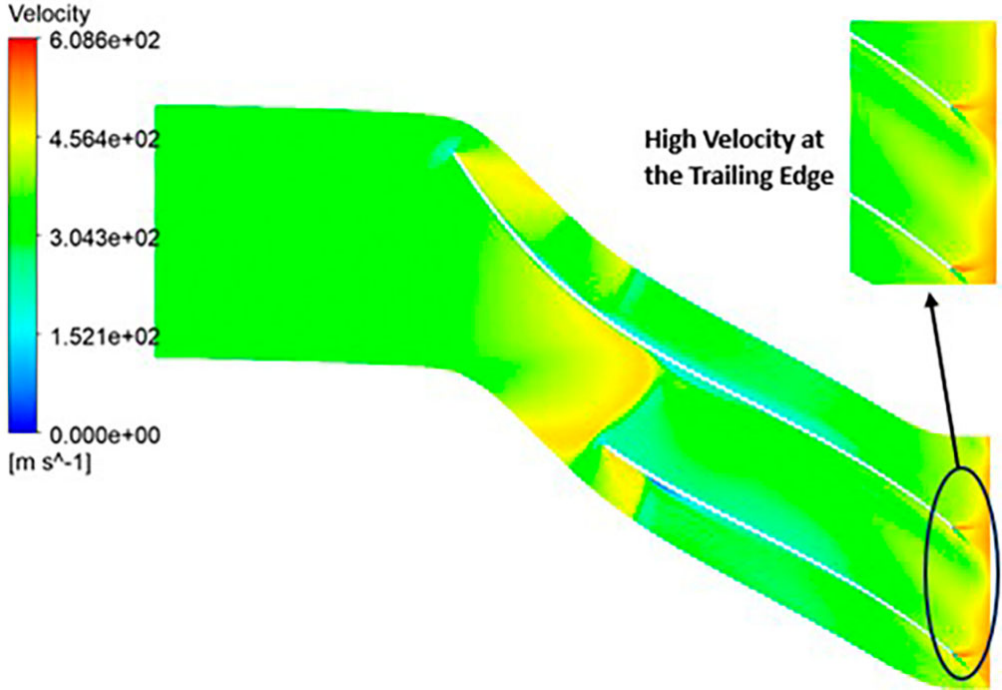
Velocity flow contour for the modified blade (k-Epsilon).

**Figure 52.**
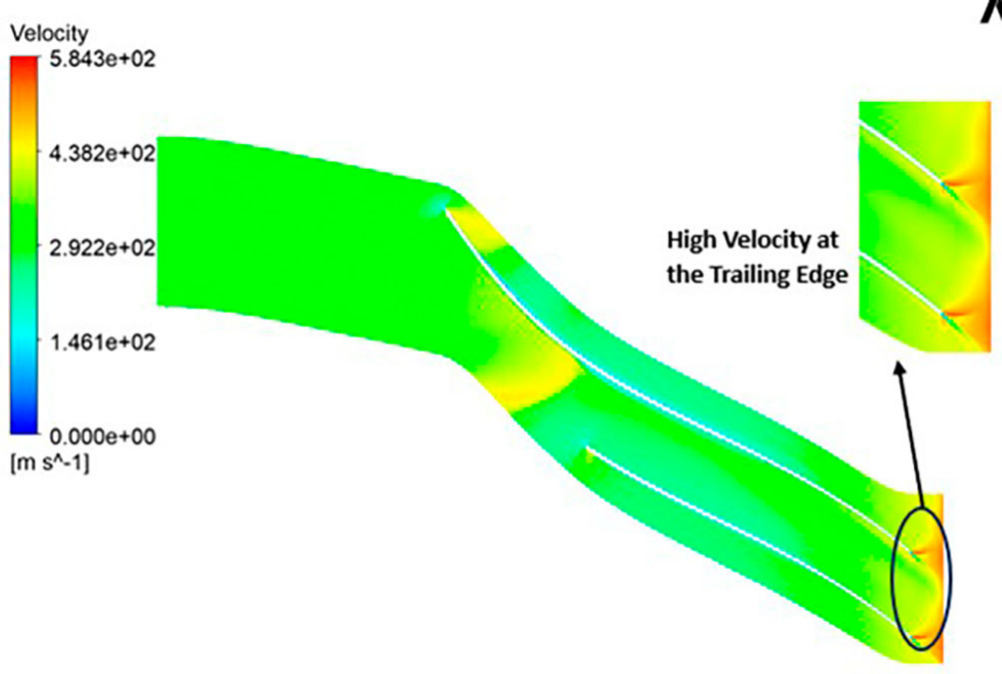
Velocity flow contour for the optimized blade (SST).

**Figure 53.**
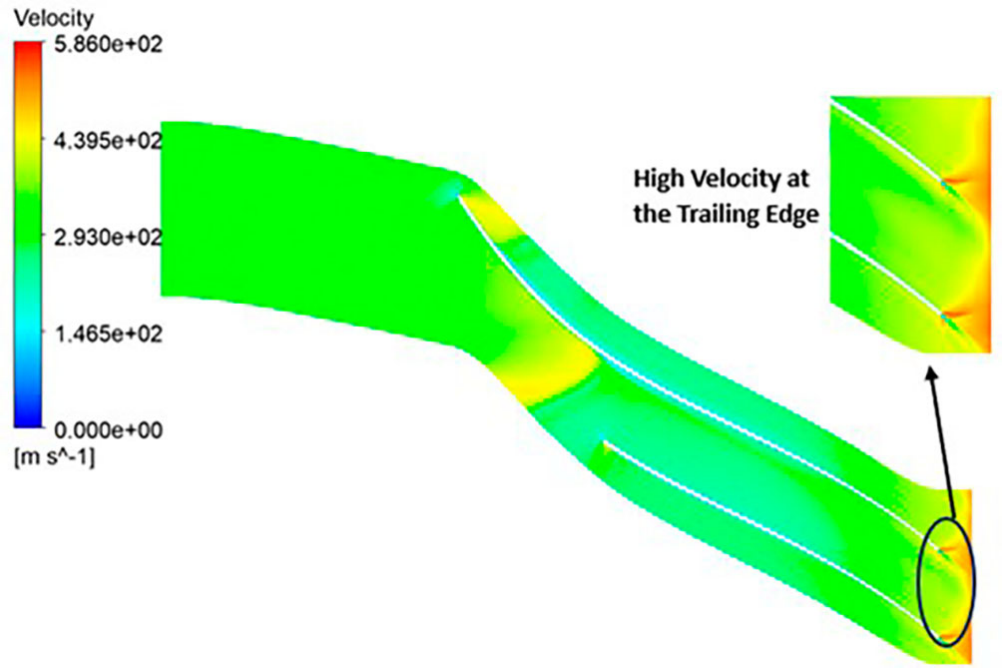
Velocity flow contour for the optimized blade (k-Epsilon).

VELOCITY: The analysis results highlight that the implementation of the optimized blade design leads to noteworthy enhancements in fluid velocity compared to the original configuration. The optimized blades effectively reduce the fluid’s velocity due to their enhanced capacity for energy transfer, resulting in a commendable reduction in fluid velocity that remains well within acceptable operational limits. Notably, distinct contours representing elevated velocity are prominently observed at the trailing edge. Across the entirety of the impeller’s span, the velocity consistently falls within a specific range. Upon meticulous examination of Figures 48 to 53, it becomes apparent that the velocity along the blade exhibits significantly lower values in comparison to other regions within the impeller. Notably, the velocity contours remain consistent, with higher velocity ranges observed in the k-Epsilon models. During the optimization process, it became evident that increasing the blade count and decreasing the hub diameter led to a decrease in velocity.

**Figure 54.**
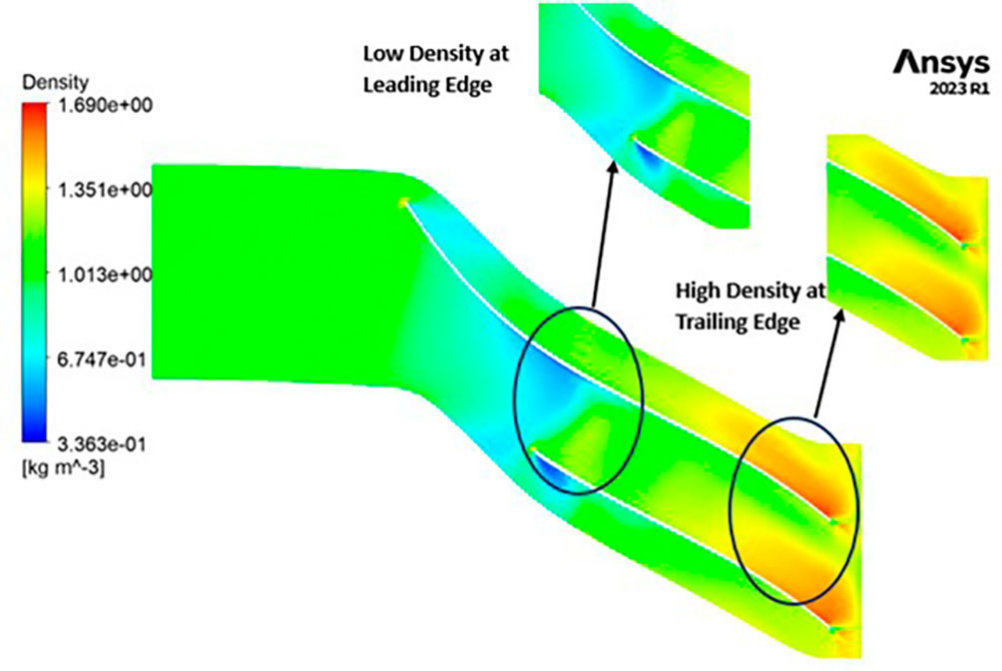
Density flow contour for baseline analysis (k-Epsilon).

**Figure 55.**
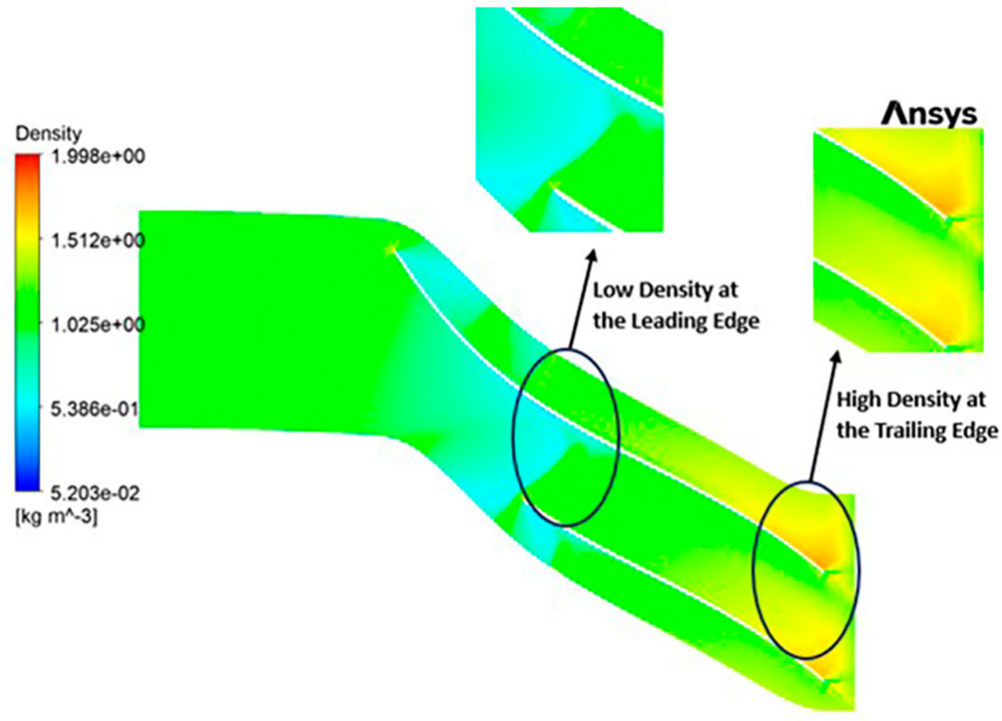
Density flow contour for the modified blade (SST).

**Figure 56.**
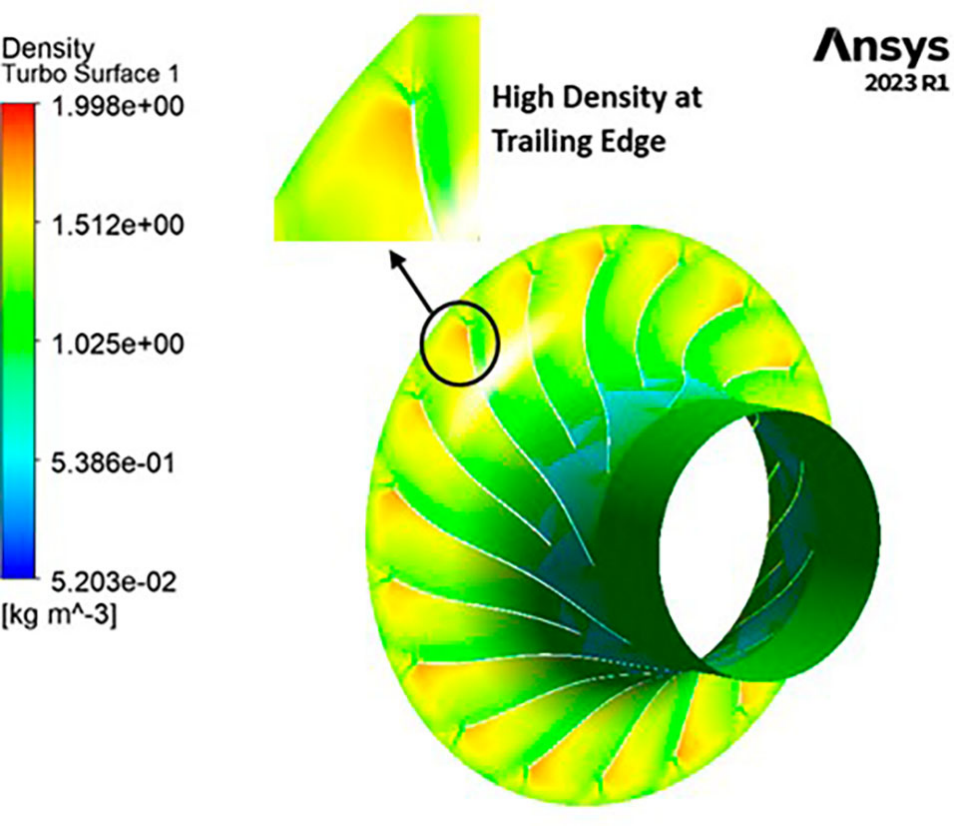
3D view density flow contour.

**Figure 57.**
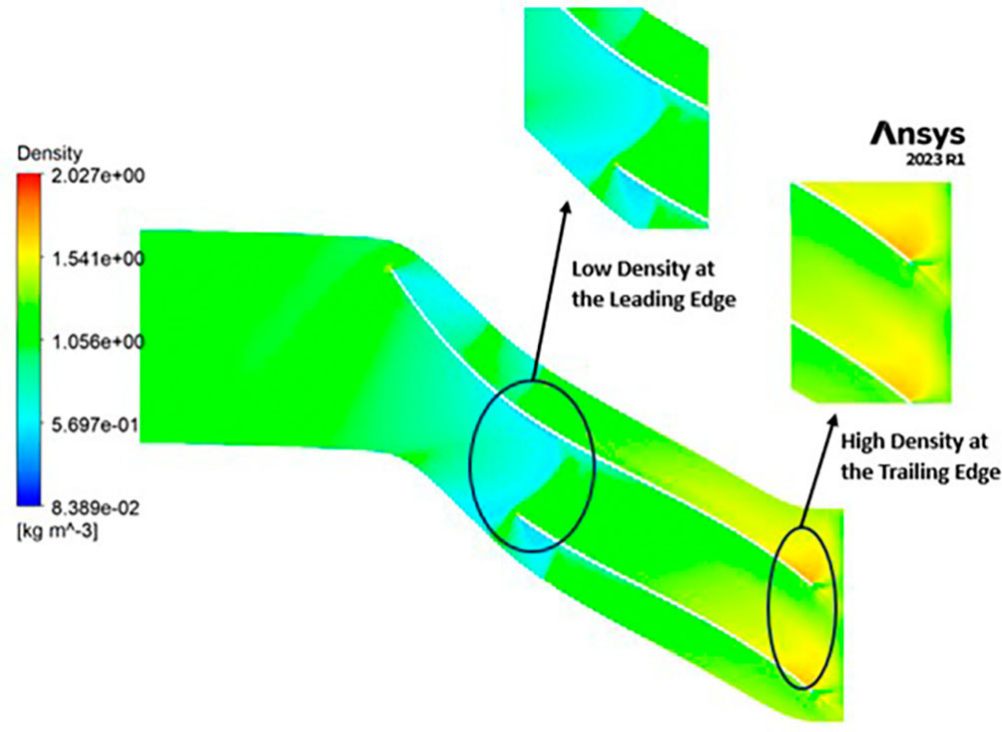
Density flow contour for the modified blade (k-Epsilon).

**Figure 58.**
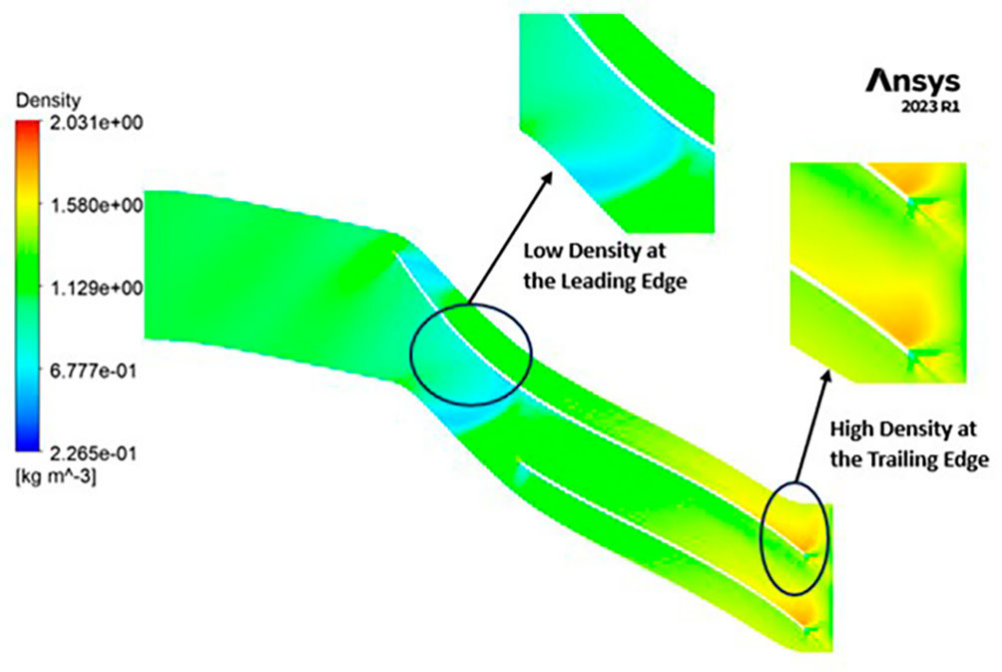
Density flow contour for the optimized blade (SST).

**Figure 59.**
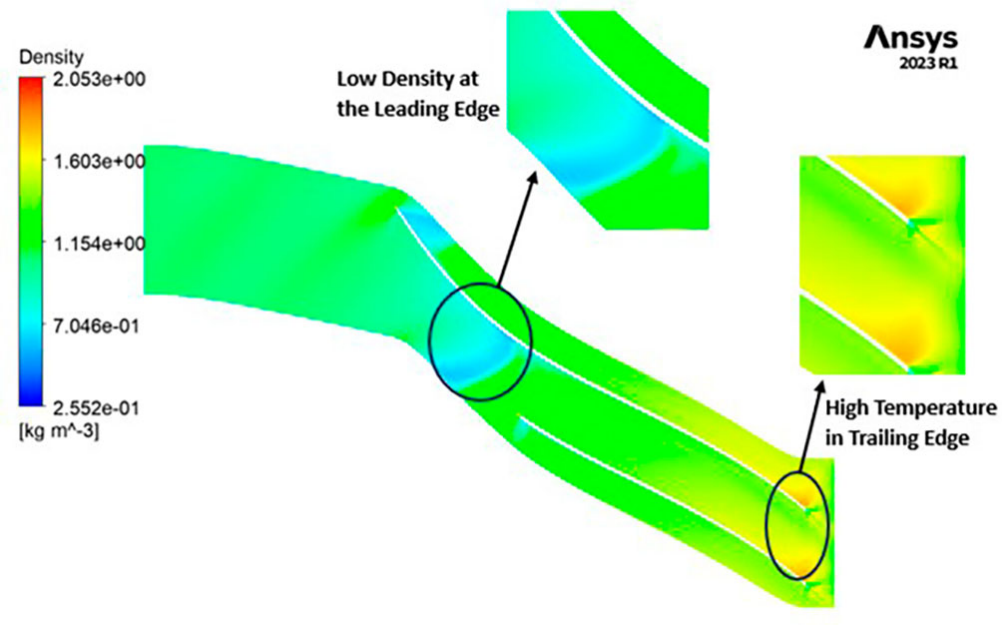
Density flow contour for the optimized blade (k-Epsilon).

DENSITY: Density assumes a central role in shaping the aerodynamic characteristics of the compressor, exerting direct influence over parameters like mass flow rate, pressure ratio, and temperature rise within the machinery. Over the course of the grid independence assessment and optimization iterations, the maximum density consistently adhered to a range spanning from 1.97 kg/m ^3^ to 2.05 kg/m ^3^. Notably, it is observed that the SST turbulence model exhibits slightly lower density values compared to the k-Epsilon turbulence model. However, the contours representing density maintain uniformity across all cases as shown in Figures 54 to 59. The quest for a homogeneous density distribution along the impeller blades remains a sought-after objective, as it fosters smoother and more predictable airflow patterns.

**Figure 60.**
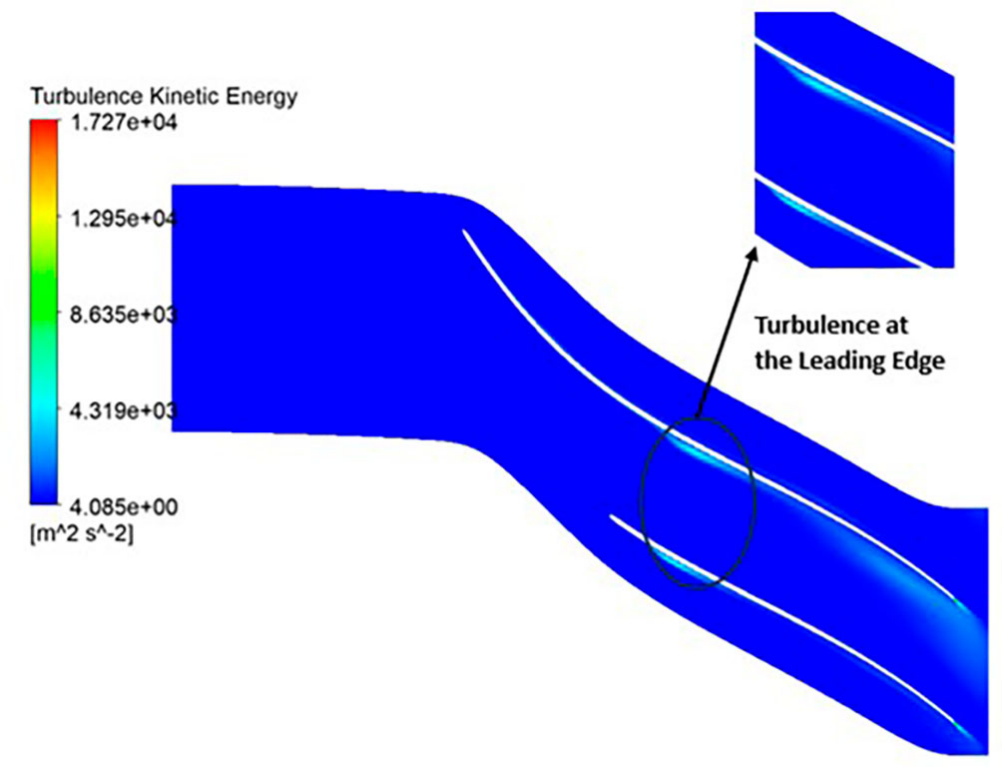
Turbulence flow contour for baseline analysis (k-Epsilon).

**Figure 61.**
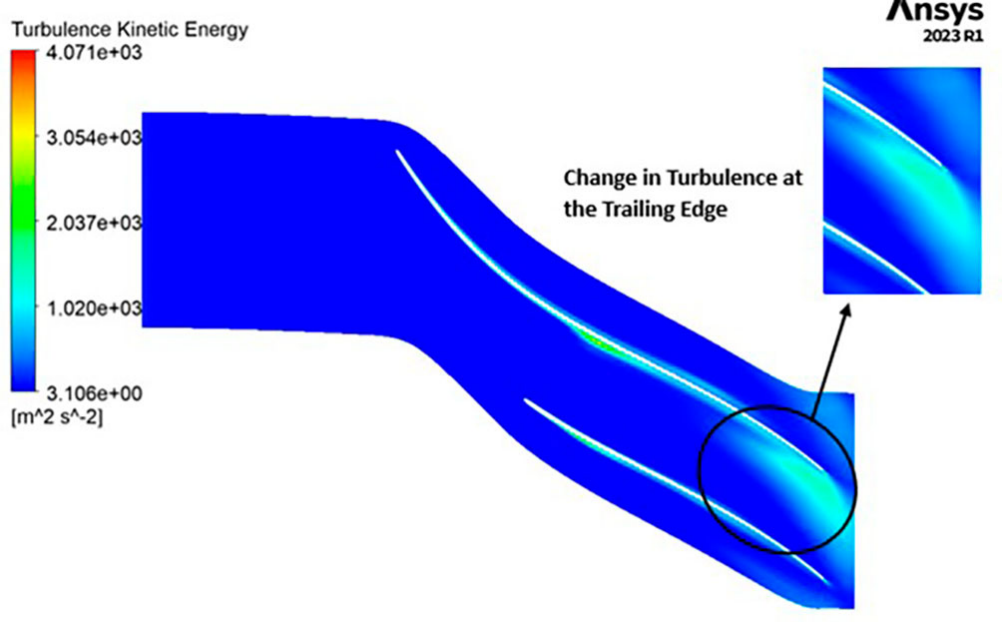
Turbulence Kinetic Energy flow contour for the modified blade (SST).

**Figure 62.**
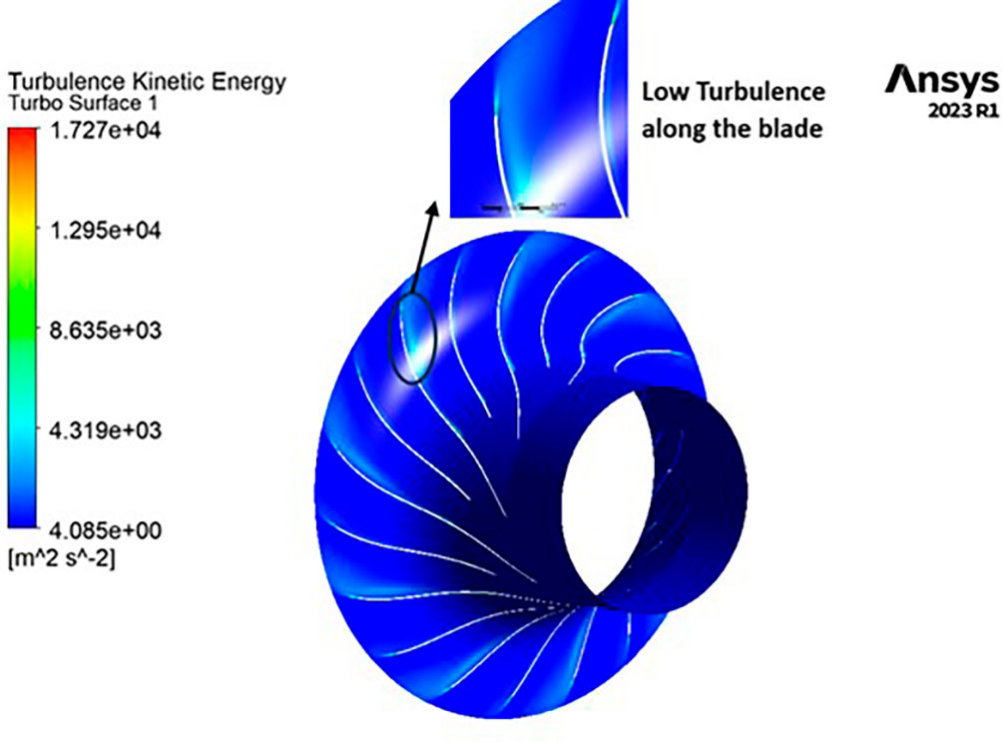
3D view turbulence kinetic energy flow contour.

**Figure 63.**
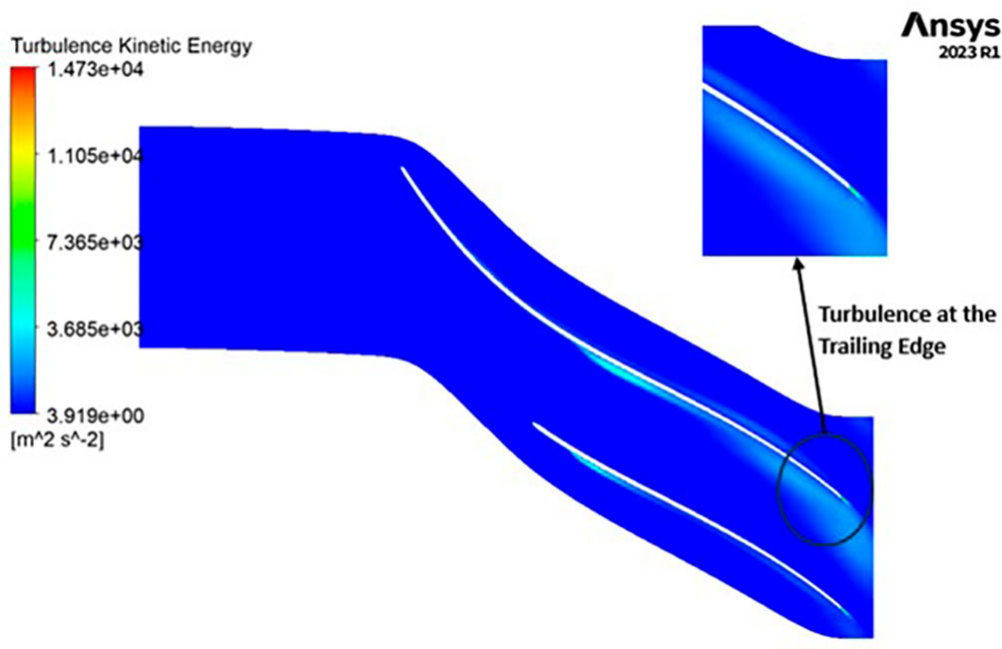
Turbulence flow contour for the modified blade (k-Epsilon).

**Figure 64.**
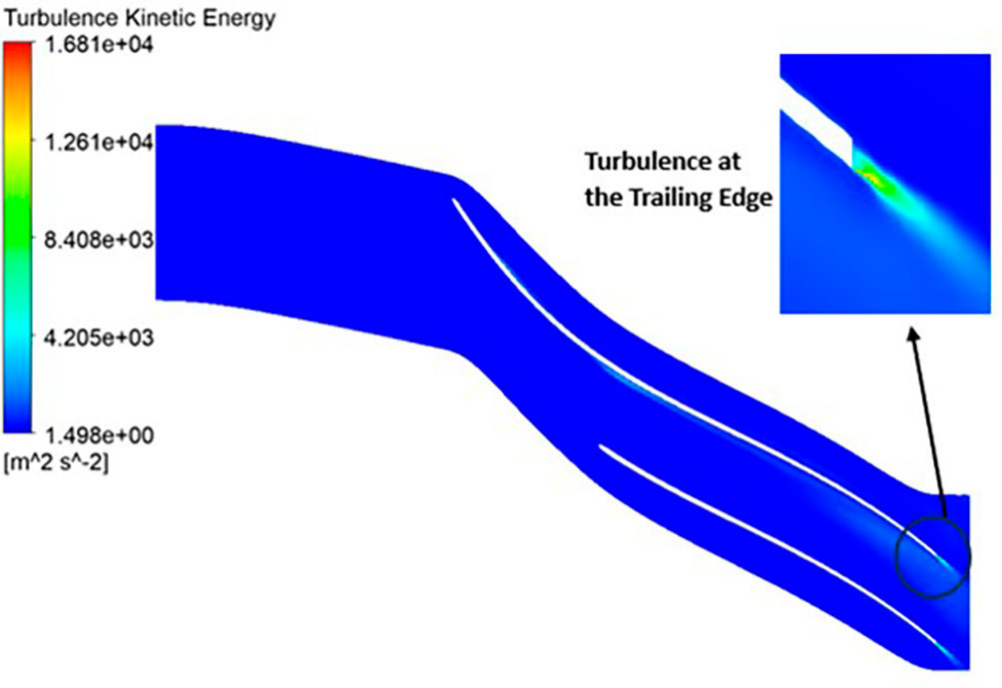
Turbulence flow contour for the optimized blade (SST).

**Figure 65.**
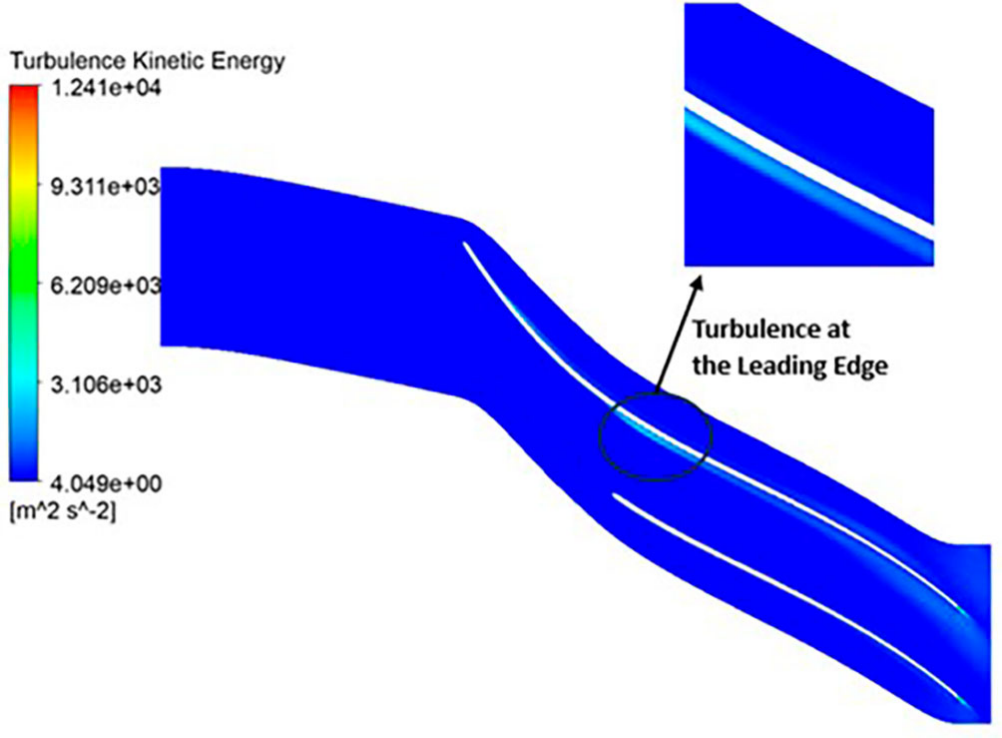
Turbulence flow contour for the optimized blade (k-Epsilon).

TURBULENCE KINETIC ENERGY: Observations indicate that the SST Turbulence model exhibits relatively higher turbulence kinetic energy levels when compared to the k-Epsilon model. In the context of the SST turbulence model applied to the modified blade, a localized region of heightened turbulence is discernible, albeit to a lesser extent in the case of the k-Epsilon model as shown in Figures 61 to 64. However, in the analyses of the optimized blade design, turbulence remains consistently low across the entire span, as depicted in Figures 60 and 65. Furthermore, turbulence levels maintain a relatively stable and subdued profile throughout the simulations. It is noteworthy that a pronounced concentration of turbulence is evident, particularly at the trailing edge of the impeller. These observations provide valuable insights into the flow characteristics and turbulence distribution within the compressor.

## Conclusion

Centrifugal compressors play a vital role in numerous industries, including air conditioning, refrigeration, gas turbines, and industrial processes. Their applications span across sectors such as air conditioning, gas turbines, oil and gas, petrochemicals, and aerospace, performing critical functions like air compression, gas transportation, and industrial processes. However, designing these compressors presents multifaceted challenges, including achieving high aerodynamic efficiency, effective surge and choke control, material selection, rotor dynamics management, cavitation mitigation, erosion prevention, and environmental compliance while optimizing costs. This research aims to comprehensively investigate and enhance the aerodynamic performance of centrifugal compressors by examining key design parameters, notably blade number and hub diameter, and evaluating various turbulence models using numerical techniques to address gaps in existing literature.

Two turbulence models, Shear Stress Transport and K-epsilon are scrutinized within the context of Computational Fluid Dynamics (CFD) simulations. CFD provides insights into the internal flow dynamics within centrifugal compressors, analysing critical variables such as Pressure, Mach Number, Density, Velocity, Turbulence Kinetic Energy, and Temperature. Despite slight discrepancies, with a 5% variance in pressure and an 18% deviation in Mach number compared to reference values, this study underscores the importance of judicious model selection and precise simulation techniques. The grid independence check confirms result stability after reaching around 5 million nodes for most properties.

A key finding relates to the correlation between the number of blades and hub diameter with the Mach number. Lower blade counts and larger hub diameters are associated with higher Mach numbers, resulting in reduced turbulence levels. Optimization identifies the configuration featuring 12 blades and a 60mm hub diameter, achieving a substantial 36% improvement in the pressure profile. Furthermore, the research highlights the cost-effectiveness of numerical simulations compared to experimental methods. It also suggests potential areas for further investigation, particularly regarding the impact of blade number and hub diameter on compressor performance. These insights, derived from rigorous scientific analysis, provide valuable guidance for engineers, manufacturers, and regulatory bodies, offering practical avenues to enhance compressor performance and efficiency in industrial applications.

## Data Availability

No data associated with this article.
