# Supplementary material for: Aerodynamic performance enhancement of centrifugal compressor using numerical techniques
Source: F1000Res. 2024 May 17;13:480. [Version 1] doi: 10.12688/f1000research.145060.1 (PMC11733421; doi:10.12688/f1000research.145060.1)
Supplement: Peer review history

# Reviewer report: Reviewer response for version 1

**DOI:** 10.5256/f1000research.158947.r356229

**Published:** 2025-01-14

**Authors:** Ajith Raj

**Affiliations:** 1 Department of Aerospace Engineering, Karunya University, Coimbatore, Tamil Nadu, India

The authors have conducted a study titled "Aerodynamic Performance Enhancement of Centrifugal Compressors Using Numerical Techniques." The findings of this research provide significant value to engineers, manufacturers, and regulatory bodies by offering critical insights and practical guidance to improve the performance and efficiency of centrifugal compressors.

 This study addresses a notable research gap in the comprehension and optimization of centrifugal compressors by examining the effects of blade number and hub diameter variations on key parameters such as pressure profiles, Mach number, and eddy viscosity.

 The methodology and results are presented with a commendable level of detail, and the percentage improvements achieved through optimization are well-aligned with expectations within this field. Still the authors can incorporate few suggestions given below before indexing.  

 1. Consider adding specific numerical or comparative data to support the claim of "36% improvement in pressure profile" for better clarity in the abstract

 2. Specifically, the reasons for flow separation observed in the pressure contours should be discussed in greater detail within the results and discussion sections.

 3. Furthermore, the grid independence check performed across varying flow parameters, including temperature, pressure, and velocity, requires elaboration to clarify the significant variations noted.

 Despite these areas for improvement, the majority of the sections are well-constructed, providing comprehensive explanations aligned with the objectives of the study.

 The paper can be accepted with these minor changes.

Is the work clearly and accurately presented and does it cite the current literature?

Yes

If applicable, is the statistical analysis and its interpretation appropriate?

Yes

Are all the source data underlying the results available to ensure full reproducibility?

Yes

Is the study design appropriate and is the work technically sound?

Yes

Are the conclusions drawn adequately supported by the results?

Yes

Are sufficient details of methods and analysis provided to allow replication by others?

Yes

Reviewer Expertise:

UAV Design and Aerodynamics, Dissimilar metal joints, Composite Materials.

I confirm that I have read this submission and believe that I have an appropriate level of expertise to confirm that it is of an acceptable scientific standard.

# Reviewer report: Reviewer response for version 1

**DOI:** 10.5256/f1000research.158947.r289525

**Published:** 2024-06-26

**Authors:** Juluru Sandeep

**Affiliations:** 1 Sandip University, Nashik, Maharashtra, India

I would like to congratulate the authors for the effort made in the research of centrifugal compressor. I recommend following suggestions to improve the quality of paper:

 1. Mention the version of ANSYS used.

 2. All the figures should be enabled with axis system (x,y,z).

 3. What is the source for Table 4 values?

 4. In Figure 28, 29 what does the profile curves with numbers 7,8,9  indicates.

 5. In the discussions change in parameter like increase or decrease is noticed but the cause for change is not clearly discussed.

Is the work clearly and accurately presented and does it cite the current literature?

Yes

If applicable, is the statistical analysis and its interpretation appropriate?

Yes

Are all the source data underlying the results available to ensure full reproducibility?

Yes

Is the study design appropriate and is the work technically sound?

Yes

Are the conclusions drawn adequately supported by the results?

Partly

Are sufficient details of methods and analysis provided to allow replication by others?

Yes

Reviewer Expertise:

My area of research is on CFD analysis of hypersonic intakes

I confirm that I have read this submission and believe that I have an appropriate level of expertise to confirm that it is of an acceptable scientific standard.
